# Mg^2+^ Catalyzes Nonenzymatic RNA Primer Extension through a Concerted Outer-Sphere Mechanism

**DOI:** 10.1021/jacs.6c06167

**Published:** 2026-06-08

**Authors:** Ruohe Wang, Collin Nisler, Qiang Cui, Laura Gagliardi, Jack W. Szostak

**Affiliations:** 1 Department of Chemistry, 2462 University of Chicago, Chicago, Illinois 60637, United States; 2 Chicago Center for Theoretical Chemistry, 2462 University of Chicago, Chicago, Illinois 60637, United States; 3 Howard Hughes Medical Institute, 2462 University of Chicago, Chicago, Illinois 60637, United States; 4 Department of Chemistry, 1846 Boston University, Boston, Massachusetts 02215, United States; 5 Department of Physics, 1846 Boston University, Boston, Massachusetts 02215, United States; 6 Department of Biomedical Engineering, 1846 Boston University, Boston, Massachusetts 02215, United States; 7 Pritzker School of Molecular Engineering, 2462 University of Chicago, Chicago, Illinois 60637, United States

## Abstract

The nonenzymatic replication of the primordial genetic material was an essential stage in the origin of life. One intensively studied model for this process is the template-directed extension of an RNA primer with imidazolium-bridged dinucleotide substrates. This reaction is catalyzed by divalent metal ions such as Mg^2+^, but the mechanism of catalysis remains poorly understood. We have utilized classical molecular dynamics together with hybrid quantum mechanics/molecular mechanics to investigate the catalytic role of Mg^2+^. We used these approaches to compute the free energy landscape along key reaction coordinates and to quantify the impact of Mg^2+^ coordination at each step of the primer extension reaction. The presence of Mg^2+^ in the reaction center significantly lowers the p*K*_a_ of the nucleophilic 3′-OH group and reduces the probability of O2′ attack. Based on the energetics of potential reaction pathways, our results suggest a preferred mechanism in which Mg^2+^ becomes outer-sphere coordinated to the oxygen of the 3′-hydroxyl group of the extending primer followed by concerted proton transfer and nucleophilic attack on the phosphate of the incoming nucleotide. Our results demonstrate the dual structural and electronic roles of Mg^2+^ in catalysis and provide insights that may inform the search for metal ion chelators that further enhance nonenzymatic primer extension.

## Graphical abstract

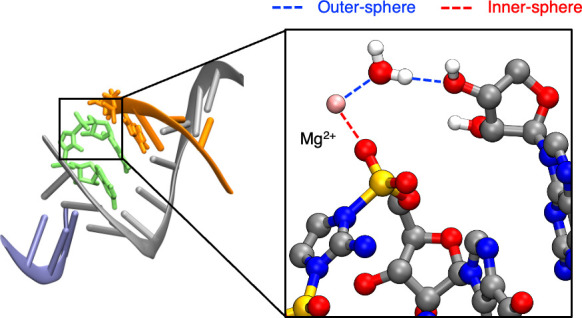

## Introduction

The origin of life on Earth is thought to have been based on RNA as the initial biopolymer, prior to the advent of protein- and DNA-based biology.^1^ The foundation of the RNA world hypothesis lies in the ability of RNA to both store genetic information and catalyze chemical reactions, including reactions related to self-replication.^2,3^ However, prior to the evolution of ribozyme catalysts, RNA replication had to rely on nonenzymatic chemical and physical processes. One model for the chemical copying of RNA templates that has been extensively explored involves primer extension with 2-aminoimidazole (2AI)-activated 5′-nucleotides. Our current understanding of this system is based on a combination of reaction kinetics, crystallography, and molecular dynamics (MD). Experimental results show that 2AI-activated ribonucleotides react with each other to form 2AI-bridged dinucleotides (N*N) that lead to efficient template copying via nonenzymatic primer extension.^4^ Interestingly, divalent metal ions such as Mg^2+^, Mn^2+^, and Fe^2+^ are crucial for efficient primer extension.^5,6^ However, a complete mechanistic understanding of the role of divalent metal ions in primer extension is lacking. Understanding the key catalytic interactions between Mg^2+^ and the RNA substrate during the reaction may lead to further advances in nonenzymatic RNA copying chemistry. For instance, the incorporation of other prebiotic molecules that enhance or strengthen desirable metal-RNA interactions may lead to increased reaction rates or improved fidelity.

Previous experimental and computational studies have begun to discover how the catalytic metal ion contributes to the mechanism of nonenzymatic RNA primer extension. In an effort to perform this reaction while protecting membrane integrity, Adamala et al. showed that citrate-chelated Mg^2+^ retained catalytic activity, suggesting that at most three coordination sites of Mg^2+^ are required for catalysis.^7^ Following this initial observation, a structural study with time-resolved X-ray crystallography was performed on a template-primer RNA duplex cocrystallized with deoxyguanosine monophosphate (dGMP).^8^ Soaking of the crystals with 2AI-activated guanosine (*G) displaced dGMP from the template, and resulted in the subsequent *in situ* formation of G*G bound to the template next to the primer. Subsequent addition of Mg^2+^ initiated the primer-extension reaction in the crystal. However, the metal-RNA interaction in the reaction center could not be seen, most likely due to the weak binding of the divalent metal ion to the reaction center and the difficulty of distinguishing Mg^2+^ from water molecules in electron density maps. A metal ion rescue study was conducted by Fang et al.^6^ as an alternative method to probe the interaction between Mg^2+^ and the imidazolium-bridged dinucleotide substrate (N*N). Two phosphorothioate diastereomers were synthesized, each with one of the two nonbridging oxygen atoms in the reactive phosphate group of the N*N substrate substituted with a sulfur atom. The reaction rates of the two diastereomers were determined and compared to that with the native substrate, in the presence of thiophilic Cd^2+^ and oxophilic Mg^2+^ divalent cations. This work confirmed a direct interaction between Mg^2+^ and the nonbridging pro-*S*_P_ oxygen of the reactive phosphate group during catalysis (Figure 1A). Mittal and Nisler et al. employed MD simulations to explore the consequences of different initial placements of Mg^2+^ in the reaction center.^9^ When Mg^2+^ forms a direct interaction with the deprotonated 3′-OH group of the attacking primer and the pro-*S*_P_ oxygen, the reactant geometry is preorganized in a manner that favors the reaction.

**Figure 1:**
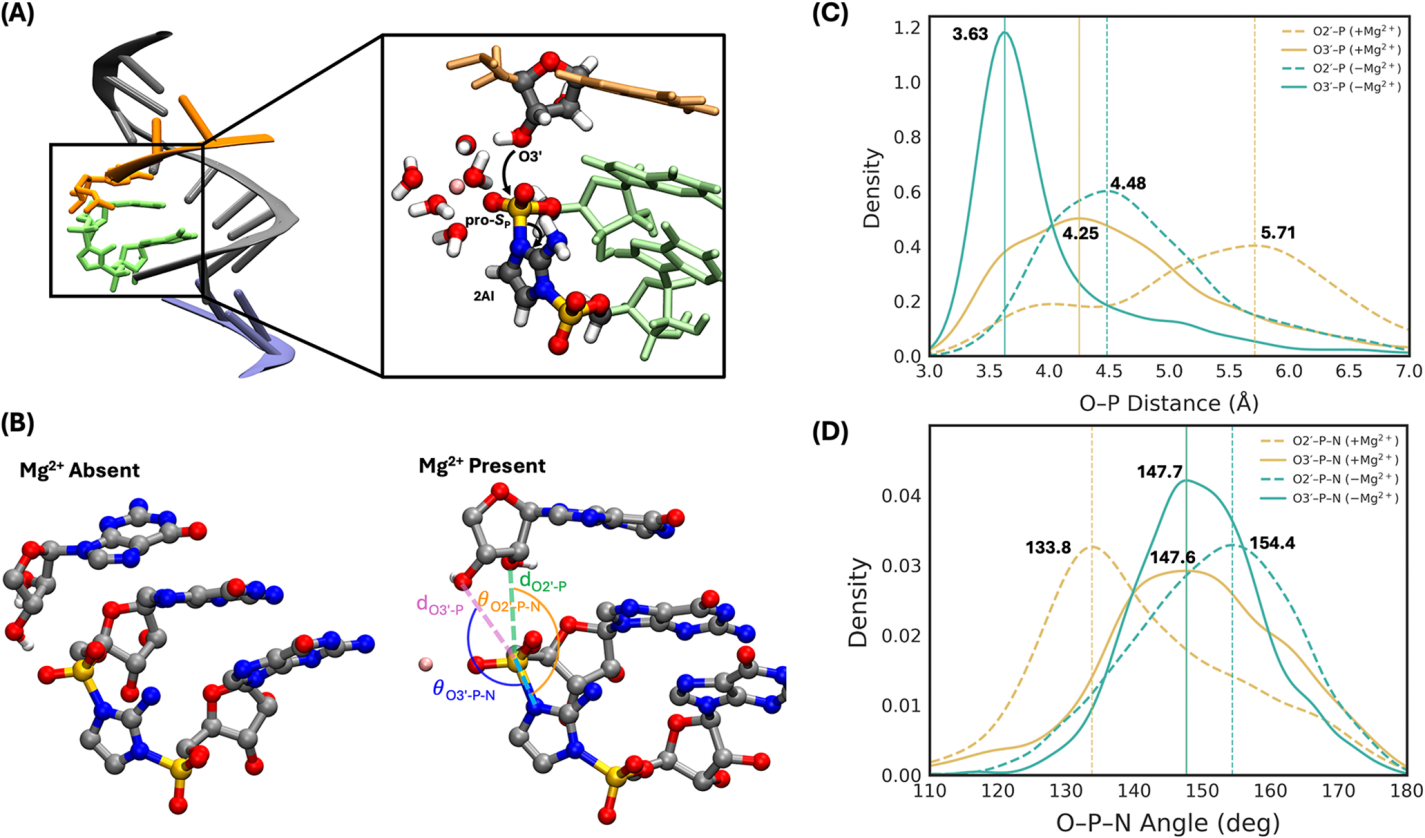
Overview of the nonenzymatic primer-extension reaction. (A) The RNA duplex consisting of the attacking primer (orange), 2-aminoimidazolium-bridged dinucleotide (G*G; green), downstream helper oligonucleotide (purple), and template strand (gray). The QM region is shown to the right with the ball-and-stick model. (B) A representative structure is shown for the attacking primer without (left) and with (right) Mg^2+^ bound to the pro-*S*_P_ oxygen in the reactive phosphate group with the OL3 nucleic acid force field, TIP4P-Ew water model, and m12–6–4 ion parameter set. The O2′–P distance (green), the O3′–P distance (purple), the O2′–P–N angle (orange), and the O3′–P–N angle (blue) are labeled. The hydrogen atoms are omitted for clarity except in the 2′-OH and 3′-OH group of the attacking nucleotide, which is within H-bonding distance of the pro-*S*_P_ phosphate oxygen. The C, H, N, O, P, and Mg atoms are colored gray, white, blue, red, orange, and pink, respectively. (C) The distribution of the O2′–P (dashed) and O3′–P (solid) distances with (beige; +Mg^2+^) and without (teal; −Mg^2+^) Mg^2+^ bound to the pro-*S*_P_ oxygen from classical MD simulations. (D) The distribution of the O2′–P–N (dashed) and O3′–P–N (solid) angles with (beige; +Mg^2+^) and without (teal; −Mg^2+^) Mg^2+^ bound to the pro-*S*_P_ oxygen from classical MD simulations. Vertical lines represent the most probable value corresponding to each distribution.

Despite this progress, major uncertainties remain concerning the interactions of the catalytic Mg^2+^ in the reaction center along the reaction pathway. Whether the Mg^2+^ interacts with the 3′-OH by inner-sphere or outer-sphere coordination, and whether deprotonation of the 3′-OH nucleophile occurs before or concerted with nucleophilic attack is still unknown. Here, we have addressed these mechanistic questions by modeling an RNA primer/template/bridged-intermediate complex (Figure 1A) using classical and hybrid quantum mechanical/molecular mechanical (QM/MM) simulations, as well as cluster models using density functional theory (DFT) and the wavefunction-based coupled cluster (CC) theory. Our results show that the presence of Mg^2+^ bound to the pro-*S*_P_ oxygen induces a pre-reaction geometry (**Pre**) that favors the desired 3′-5′ extended product and reduces the p*K*_a_ of the 3′-OH group. By performing QM/MM and DFT calculations, we have quantified and compared the energetics of different potential reaction mechanisms, beginning with Mg^2+^ bound to the pro-*S*_P_ oxygen. We modeled the reaction pathway starting from a reactant state with a protonated 3′-OH group and a metal-bound hydroxide ion (**React**), transitioning to an intermediate state with a deprotonated 3′-OH (**Int**), and then to a product state after the nucleophilic attack of O3′ on the P atom in the reactive phosphate group (**Prod**; Scheme 1). Along this path, the catalytic Mg^2+^ can change its binding mode to adjacent ligands of the substrate, including the pro-*S*_P_ oxygen (**PO**), O3′ (**3′O**), and the nitrogen atom of the bridging group (**N**), between direct inner-sphere coordination (denoted as **IS**) and indirect outer-sphere coordination mediated by a bridging water molecule (denoted as **OS**).^10^ These binding mode changes were incorporated into our calculations, providing an exhaustive characterization of potential mechanistic pathways for Mg^2+^-catalyzed primer extension and revealing a preference for a concerted outer sphere reaction mechanism. These resulting atomistic insights expand our understanding of the nonenzymatic primer-extension reaction and will also inform future experimental and computational efforts to design chelators that improve metal ion catalysis.

**Scheme 1:**
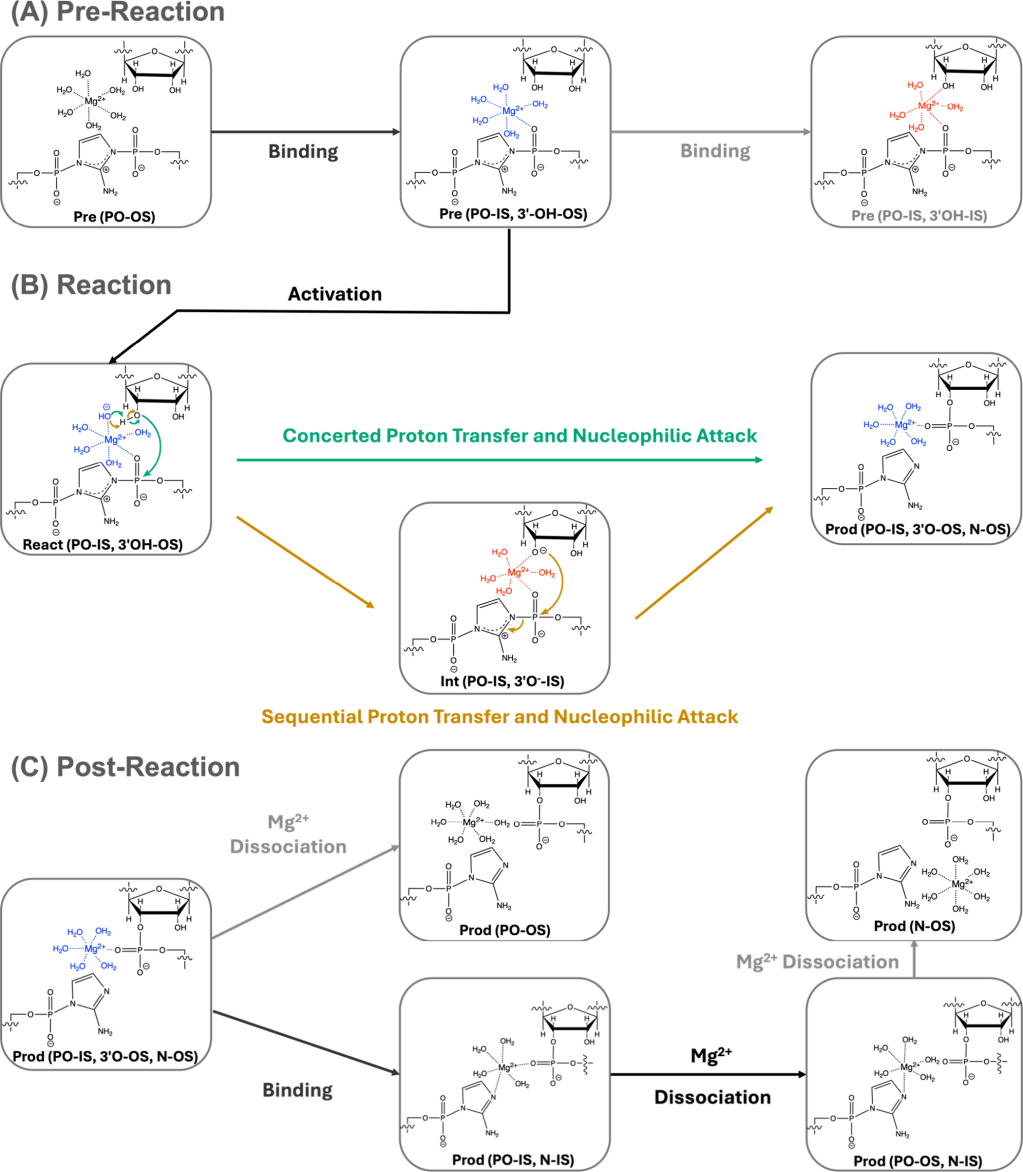
Proposed Mechanisms for Nonenzymatic RNA Primer Extension for the Pre-reaction (A), Reaction (B), and Post-reaction (C) Stages^a^

a Pre-reaction (Pre), reactant (React), deprotonated intermediate (Int), and product (Prod) states are illustrated. The inner-sphere (IS) and outer-sphere (OS) Mg^2+^–O3′ coordination are colored as red and blue, respectively. The concerted mechanism (teal; React (PO-IS, 3′OH-OS) ***→*** Prod (PO-IS, 3′O-OS)) and stepwise mechanism (beige; React (PO-IS, 3′OH-OS) ***→*** Dep (PO-IS, 3′O^–^-IS) ***→*** Prod (PO-IS, 3′O-OS)) are shown with colored arrows. PO refers to the pro-*S*_P_ oxygen of the reacting phosphate. The off-path steps are denoted with gray arrows.

## Results

### Structural Effects of Mg^2+^ Coordination to the Reaction Center

To determine the effect that Mg^2+^ binding has on the structural dynamics of the nonenzymatic primer extension reaction center, we performed classical MD simulations (Figure 1A). We used RESP-derived charges for the 2AI-bridged dinucleotide (Table S1) and force field parameters for the bridging group from the AMBER force field of similar motifs. The relative orientation of the 2AI bridging group with this parametrization remains stable and agrees with the crystal structure and previous simulations (Figure S1).^9,11^

Previous experimental and computational results are consistent with a direct inner-sphere coordination of the catalytic Mg^2+^ atom with the pro-*S*_P_ oxygen. We consider this interaction to represent the first step of catalysis.^6,9^ To investigate the structural effects induced by this binding, we ran classical equilibrium MD simulations on the primer/template/bridged-intermediate complex in the absence and in the presence of this Mg^2+^: pro-*S*_P_ oxygen interaction (Figure 1B), with a protonated 3′-OH. Both systems were pre-equilibrated for 100 ns, followed by a 200-ns production run (Sim1 and Sim2; Table S2). Two collective variables were selected for comparison because of their expected effect on reactivity: the distance between O3′ and the P atom in the reactive phosphate group, since phosphodiester bond formation involves a decrease in O3′–P distance, and the angle of attack between O3′, P, and N of the 2AI bridging group (Figure 1), where an angle closer to 180° is favorable for in-line attack. In the absence of the catalytic Mg^2+^ ion, the O3′–P distance distribution over the entire simulation has a mean of 4.00 ± 0.72 Å with a most probable value of 3.63 Å, while the O3′–P–N angle distribution has a mean of 149.6 ± 9.6° with a most probable value of 147.7° (Figure 1C,D, solid teal lines). A stable hydrogen bond between the 3′-OH and the pro-*S*_P_ oxygen of the reactive phosphate is also observed (Figure S2). Surprisingly, when Mg^2+^ is bound to the pro-*S*_P_ oxygen, the O3′-P distance distribution shifts to a larger mean value of 4.60 ± 0.88 Å, with the most probable value of 4.25 Å, while the O3′–P–N angle distribution shifts to a slightly smaller mean value of 148.4 ± 13.7°, with a most probable value of 147.6° (Figure 1C,D, solid beige lines). This suggests that, when the primer O3′ is protonated, Mg^2+^ reduces the reactivity of the primer extension complex. We also analyzed the difference between the O2′–P and O3′–P distances, and between the O2′–P–N and O3′–P–N angles. The most probable O3′–P distance is 0.85 Å and 1.46 Å shorter than the O2′–P distance without and with the bound Mg^2+^, respectively (Figure 1C). In addition, while the bound Mg^2+^ changes the most probable O3′–P–N angle by only 0.1°, it decreases the most probable O2′–P–N angle by 20.6° (Figure 1D). These results suggest that while Mg^2+^ does not have a significant effect on the 3′–5′ reactive geometry when O3′ is protonated, it more strongly disfavors geometries associated with attack by the 2′-OH group.

### Mg^2+^ Lowers the p*K*_a_ of the Primer 3′-OH Group

The presence of a divalent ion such as Mg^2+^ in the reaction center will have significant electrostatic effects on its environment. The p*K*_a_ of the cis-diol of nucleosides has been measured experimentally to be approximately 12–13, but the presence of a negatively charged 5′-phosphate should raise this p*K*_a_.^12–16^ Thus, assuming a similar p*K*_a_ for the 2′- and 3′-OH groups in the cis-diol, the 3′-OH group has a minimum p*K*_a_ of around 12–13. Since the protonated 3′-OH is a poor nucleophile, deprotonation must precede or happen concurrently with nucleophilic attack on the reactive phosphate. The presence of the bound Mg^2+^ has been predicted to decrease the 3′-H p*K*_a_, thereby facilitating its deprotonation. To test this computationally, we investigated how the binding of Mg^2+^ to the pro-*S*_P_ phosphate oxygen would affect the thermodynamics of the 3′-OH deprotonation in the attacking primer using thermodynamic integration (Figure S3). To model deprotonation, the proton of the 3′-OH group is annihilated along with a Cl^–^ counterion to maintain a neutral overall charge of the system. The difference between the calculated free energy changes for this process with and without Mg^2+^ bound to the pro-*S*_P_ oxygen corresponds to the p*K*_a_ shift induced by Mg^2+^ binding and does not assume the identity of the proton acceptor in the microscopic reaction pathway. This calculated difference was 4.3 ± 0.1 kcal/mol, corresponding to a decrease in p*K*_a_ of 3.2 ± 0.1 units. These results support the hypothesis that binding of Mg^2+^ to the pro-*S*_P_ phosphate oxygen would lower the p*K*_a_ of the reactive 3′-OH to approximately 9–10, priming it for deprotonation and nucleophilic attack. Our calculations are consistent with prior experimental data in which the rate of the primer-extension reaction exhibits an apparent p*K*_a_ of about 9.^16^

### Movement of Mg^2+^ from Bulk Solution to Inner-Sphere Coordination to the pro-*S*_P_ Oxygen

We considered that hexa-aquo Mg^2+^ in the bulk solution would likely first engage in an outer-sphere interaction with the pro-*S*_P_ oxygen and then transition to a direct inner-sphere coordination to the pro-*S*_P_ oxygen. We refer to the pre-reaction state where Mg^2+^ in the bulk solution has become outer sphere coordinated to the pro-*S*_P_ oxygen as state **Pre (PO-OS)**, and to the subsequent inner-sphere coordinated state as **Pre (PO-IS)** (see Scheme 1A).

To model the transition from outer- to inner-sphere coordination, we took coordinates from a simulation in which the catalytic Mg^2+^ spontaneously unbinds from the pro-*S*_P_ oxygen. Classical umbrella sampling was performed by splitting this trajectory into evenly spaced distances between Mg^2+^ and the pro-*S*_P_ oxygen, ranging from 1.9 to 4.8 Å. Starting from **Pre (PO-OS)**, Mg^2+^ can then become inner sphere coordinated to the pro-*S*_P_ oxygen, state **Pre (PO-IS)** (Figure 2A). We observe local energy minima at 2.0 and 4.3 Å for the inner- and outer-sphere coordinated states, with the inner-sphere coordinated state lying 4.7 kcal/mol higher in energy compared to the outer-sphere coordinated state. The forward and backward barriers to movement from the outer- to inner-sphere coordination are 13.8 and 9.1 kcal/mol, respectively. These barriers are consistent with the equilibrium MD simulations, in which repeated binding-unbinding events were not observed on the simulation time scale (Figure S4), suggesting that the inner- and outer-sphere states are kinetically trapped. The positive calculated free energy change for inner-sphere coordination of the Mg^2+^ to the pro-*S*_P_ oxygen also aligns with the very high *K*_m_ for Mg^2+^ determined from reaction kinetics.^6^ Although inner-sphere binding should be electrostatically favored, we suggest that the interaction is energetically disfavored due to entropic effects including localization of the Mg^2+^ ion and rigidification of the surrounding RNA structure, as well as loss of 3′OH–O­(pro-*S*_P_) hydrogen bond.

**Figure 2:**
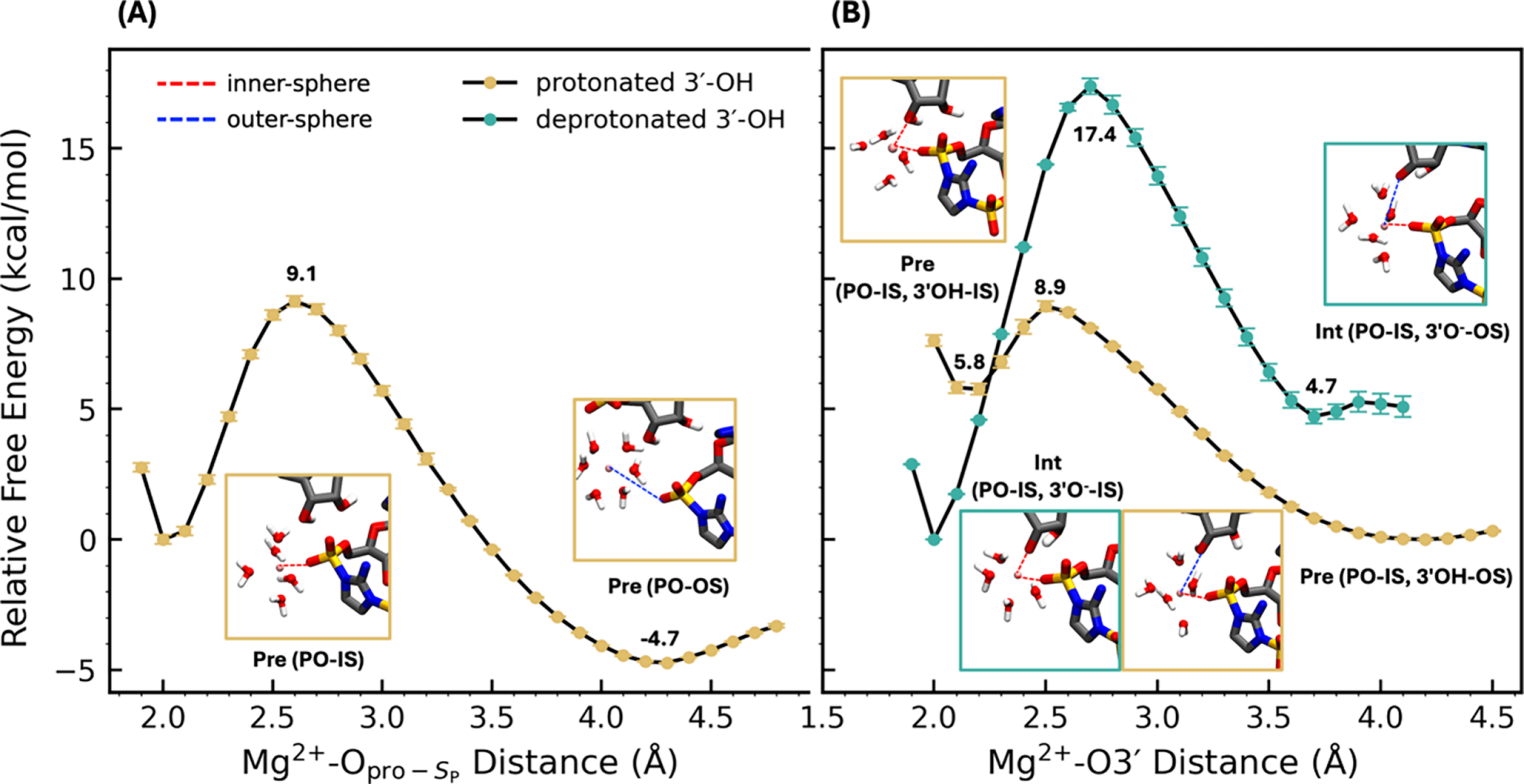
Potential of mean force as a function of the distance between Mg^2+^and the pro-*S*_P_ oxygen and O3′ using classical umbrella sampling. (A) The initial state **Pre (PO-OS)** corresponds to the outer-sphere coordination of Mg^2+^ to the pro-*S*_P_ oxygen. As the Mg^2+^ approaches the pro-*S*_P_ oxygen, the system transitions to state **Pre (PO-IS)**, in which the Mg^2+^ is inner sphere coordinated to the pro-*S*_P_ oxygen. (B) Once bound to the pro-*S*_P_ oxygen, Mg^2+^ can then transition to the outer-sphere coordination to the protonated 3′-OH, defined as state **Pre (PO-IS, 3′OH-OS)**, which is energetically uphill by 5.8 kcal/mol compared to the inner-sphere coordination, defined as state **Pre (PO-IS, 3′OH-IS)**. Alternatively, deprotonation of the 3′-OH converts state **Pre (PO-IS, 3′OH-OS)** to state **Int (PO-IS, 3′O** ^**–**^ **-OS)**. The transition to inner-sphere coordination of the Mg^2+^ to the 3′-O^–^, i.e., state **Int (PO-IS, 3′O** ^**–**^ **-IS)**, is energetically favorable by 4.7 kcal/mol. State labels are as in Scheme 1A,B. Beige: protonated 3′-OH. Teal: deprotonated 3′-O^–^. Error bars are the standard error of the mean from three replicates. The states **Pre (PO-IS, 3′OH-OS)** and **Int (PO-IS, 3′O** ^**–**^ **-IS)** are aligned to zero and are not directly comparable. Representative structures corresponding to the minimum energy are shown. The hydrogen atoms are omitted for clarity except in water and the 2′-OH and 3′-OH group of the attacking nucleotide.

### Interaction of the Bound Mg^2+^ with the Primer O3′

Because the 3′-OH of the terminal nucleotide of the primer presents a likely site of contact with the Mg^2+^ bound to the pro-*S*_P_ oxygen, we explored possible binding modes between Mg^2+^ and O3′ with classical MD. The initial coordinate featured a protonated 3′-OH group and inner-sphere Mg^2+^–O3′ coordination, denoted as **Pre (PO-IS, 3′OH-IS)**. During the 100-ns equilibration, the Mg^2+^–O3′ coordination spontaneously switched to the more stable outer-sphere coordination, denoted as **Pre (PO-IS, 3′OH-OS)** (see Sim1 in Table S2).

We then investigated the binding mode of the Mg^2+^ ion when the 3′-OH was deprotonated, which we refer to as the intermediate state (**Int**). To do this, the proton of the 3′-OH group was removed from an equilibrated snapshot of the Sim1 simulation to generate the state **Int (PO-IS, 3′O** ^**–**^ **-OS)** (see Sim3 in Table S2). RESP charges were used for the attacking nucleotide with a 3′-O^–^ alkoxide (). In this case, the catalytic Mg^2+^ moves spontaneously to become inner sphere coordinated to the 3′O^–^, while remaining inner sphere coordinated to the pro-*S*_P_ phosphate oxygen (**Int (PO-IS, 3′O** ^**–**^ **-IS)**). In contrast to the preferred outer-sphere coordination to the protonated 3′-OH, inner-sphere coordination is favored after 3′-OH deprotonation.

To quantify the energetics of these changes in binding mode, we performed umbrella sampling using the Mg^2+^–O3′ distance as the collective variable (Figure 2B). Initial coordinates for the Mg^2+^–O3′ distances that were not well sampled were taken from snapshots of adjacent windows near equilibrium. When the 3′-OH group is protonated, the inner- (**Pre (PO-IS, 3′OH-IS)**) and outer-sphere (**Pre (PO-IS, 3′OH-OS)**) coordinated complexes reach their energetic minima at Mg^2+^–O3′ distances of 2.2 Å and 4.2 Å, respectively (Figure 2B). There is a barrier of 8.9 kcal/mol to move from outer-sphere coordination to inner-sphere coordination, with the outer-sphere state lying 5.8 kcal/mol lower in energy. The barrier to move from inner- to outer-sphere coordination of 3.1 kcal/mol is relatively low. Thus, Mg^2+^ bound to the pro-*S*_P_ oxygen prefers to bind by outer-sphere coordination to the protonated 3′-OH group. After deprotonation, the local energy minima corresponding to the inner- (**Int (PO-IS, 3′O** ^**–**^ **-IS)**) and outer-sphere (**Int (PO-IS, 3′O** ^**–**^ **-OS)**) coordination are found at Mg^2+^–O3′ distances of 2.0 Å and 3.7 Å, respectively (Figure 2B). Contrary to the protonated state, the inner-sphere coordination lies 4.7 kcal/mol lower in energy than the outer-sphere coordination. The forward and reverse barriers for conversion from the inner- to outer-sphere coordination become 17.4 and 12.7 kcal/mol, respectively, which is notably higher than for the protonated state. As suggested by the equilibrium simulations, the umbrella sampling results quantitatively demonstrate that the favored binding mode for the bound Mg^2+^ is outer-sphere coordination to the protonated 3′-OH group, but inner-sphere coordination upon 3′-OH deprotonation. This is consistent with the previous results by Mittal and Nisler et al.^9^

The differing preferred binding modes with the pro-*S*_P_ oxygen and the protonated and deprotonated O3′ likely reflect a balance between enthalpic and entropic contributions. While the positively charged Mg^2+^ is electrostatically attracted to the negatively charged oxygen atoms, the entropic penalty of forming a more ordered inner-sphere complex can only be compensated when the oxygen carries sufficient negative charge, as in the deprotonated 3′-OH group.

### Potential Reaction Pathways for Phosphodiester Bond Formation

The Mg^2+^-catalyzed nonenzymatic RNA primer-extension reaction could potentially proceed via alternative mechanistic steps (Scheme 1B). To identify the most favorable pathway, we quantified the dynamics and energetics of each step under different Mg^2+^–O3′ binding modes. To achieve a balance between computational cost and accuracy, we employ a QM/MM scheme that has been used in mechanistic studies of biological systems with metal cofactors, including enzymes^17,18^ and ribozymes.^19,20^ In our system, the QM region consists of 52 atoms near the reaction center (Figure 1A) and is treated at the DFT level of theory, while the rest of the system is treated using a classical force field (see Methods).

Based on thermodynamic integration, Mg^2+^ bound to the pro-*S*_P_ oxygen lowers the p*K*_a_ of the 3′-OH group. To model the explicit proton-transfer step in the QM/MM simulations, we consider a Mg^2+^-bound hydroxide as the immediate proton acceptor for the 3′-OH group. This modeling choice is also consistent with previous experimental observations that the reaction rate varies with the identity of the metal ion and correlates with the p*K*_a_ value of the corresponding metal-aquo complex.^5,6^ Formation of Mg^2+^-bound hydroxide requires prior deprotonation of a Mg^2+^-bound water (**Pre (PO-IS, 3′OH-OS)** → **React (PO-IS, 3′OH-OS)**). Because Mg^2+^ is coordinated to the negatively charged pro-*S*_P_ oxygen, the p*K*_a_ of the Mg^2+^-bound water is likely higher than that of the hexaaquo complex in solution (11.2–11.4).^21^ As an approximation, we use the latter to obtain a lower-bound estimate of around 4 kcal/mol for the free-energy cost of generating Mg^2+^-bound hydroxide at the experimental pH of 8.5. To explore these potential states, we performed adaptive steered MD (ASMD) simulations with several reaction coordinates, including proton transfer, nucleophilic attack, and the Mg^2+^–O3′ distance.^22,23^ We used the pulling work to estimate the potential of mean force in each chemical step as summarized in Table S3.

#### Proton Transfer

Starting from **React** state, in which the Mg^2+^ ion is coordinated to a hydroxide ion, inner sphere coordinated with the pro-*S*_P_ phosphate oxygen **(PO-IS)**, and outer sphere coordinated with the protonated primer 3′-OH, **(3′OH-OS)** (Scheme 1B), the proton transfer step was modeled by steering either the Mg^2+^–O3′ distance or the proton transfer coordinate separately or concertedly. When only the Mg^2+^–O3′ distance is steered to change from outer- to inner-sphere coordination **React (PO-IS, 3′OH-OS)** *→* **React (PO-IS, 3′OH-IS)**, proton transfer from the 3′-OH to the Mg^2+^-bound hydroxide occurs spontaneously (Figure S5). Notably, the change in Mg^2+^ coordination and protonation state is highly thermodynamically unfavorable (by 16 kcal/mol). However, this state lies in a shallow energy well, and could have a kinetically significant lifetime.

When steered only along the proton transfer coordinate, the deprotonated 3′-O^–^ is immediately reprotonated by another Mg^2+^-bound water molecule (Figure S6). We denote this new state, in which the metal-bound hydroxide ion is in a different relative position as **React (PO-IS, 3′OH-OS)′**. Deprotonation of the 3′-OH while maintaining outer sphere coordination to the metal ion is energetically uphill by about 12 kcal/mol.

Since changing the metal ion from outer- to inner-sphere coordination and changing the protonation state of the primer hydroxyl are both individually unfavorable, we decided to examine concerted proton transfer with binding mode change. As expected, this concerted change, i.e., (**React (PO-IS, 3′OH-OS)** *→* **Int (PO-IS, 3′O** ^**–**^ **-IS)**) is also highly thermodynamically unfavorable, by about 19 kcal/mol (Figure S7, and Table S3), but could be kinetically transiently stable. Altogether, the ASMD results suggest that, prior to the nucleophilic attack step, either binding mode change (**React (PO-IS, 3′OH-OS** *→* **IS)**) or deprotonation of the 3′-OH under outer-sphere coordination (**React (PO-IS, 3′OH-OS)** *→* **Int (PO-IS, 3′O** ^**–**^ **-OS)**) are unlikely events. In addition, concerted 3′-OH deprotonation with binding mode change is also thermodynamically unfavorable (by 15.7 kcal/mol) prior to the nucleophilic attack step (Table S3).

#### Nucleophilic Attack

After modeling potential deprotonation pathways, we modeled the nucleophilic attack step directly from the **React (PO-IS, 3′OH-OS)** state and from the deprotonated intermediate **Int (PO-IS, 3′O** ^**–**^ **-IS)** identified previously. We found that concerted proton transfer and nucleophilic attack while maintaining outer-sphere Mg^2+^–O3′ coordination (**React (PO-IS, 3′OH-OS)** to **Prod (PO-IS, 3′O-OS)**; Figure S8) exhibits a similar barrier height as nucleophilic attack starting from the deprotonated intermediate while maintaining the inner-sphere Mg^2+^–O3′ coordination (**Int (PO-IS, 3′O** ^**–**^ **-IS)** *→* **Prod (PO-IS, 3′O-IS)**; Figure S9). Concerted nucleophilic attack and binding mode change (**Int (PO-IS, 3′O** ^**–**^ **-IS)** to **Prod (PO-IS, 3′O-OS)**) has a lower barrier height (Figure S10), but this barrier may be underestimated because it is inferred from reverse pulling. Among these pathways, the steps that lead to the outer-sphere product state **P-OS** (i.e., **React (PO-IS, 3′OH-OS)** to **Prod (PO-IS, 3′O-OS)** and **Int (PO-IS, 3′O** ^**–**^ **-IS)** to **Prod (PO-IS, 3′O-OS)**) are exergonic, whereas the formation of the inner-sphere product via **Int (PO-IS, 3′O** ^**–**^ **-IS)** *→* **Prod (PO-IS, 3′O-IS)** is less thermodynamically favorable. The preference for the outer-sphere Mg^2+^–O3′ coordination in the product state is expected since the inner-sphere coordinated state is strained, as confirmed by the increase in free energy from **Prod (PO-IS, 3′O-OS)** to **Prod (PO-IS, 3′O-IS)** with ASMD (Figure S11).

Taken together, the results suggest that two of the pathways have qualitatively similar reaction barriers. We have therefore studied these pathways in greater detail, as described below.

### Inner- and Outer-Sphere Mg^2+^–O3′ Coordination Produce Similar Intrinsic Nucleophilic Attack Barriers

To examine the relative energetics of the stable states identified in ASMD in more detail, we constructed cluster models corresponding to the stable states identified before, including **Int (PO-IS, 3′O** ^**–**^ **-IS)**, **Prod (PO-IS, 3′O-IS)**, **React (PO-IS, 3′OH-OS)**, and **Prod (PO-IS, 3′O-OS)**, and the corresponding transition states to estimate their relative free energies at both the DFT and CC level of theory (Figure 3). To minimize the error introduced by the changing number of water molecules in the first solvation shell of Mg^2+^ during binding mode changes, only states with the same Mg^2+^–O3′ coordination mode are compared. Structural constraints were used to maintain biological relevance as described in the *Methods* section. Although the concerted mechanism (**React (PO-IS, 3′OH-OS)** *→* **Prod (PO-IS, 3′O-OS)**; 56 atoms) has one more water molecule compared to the stepwise mechanism (**Int (PO-IS, 3′O** ^**–**^ **-IS)** *→* **Prod (PO-IS, 3′O-IS)**; 53 atoms) in the cluster model due to the different Mg^2+^–O3′ coordination mode, both involve the formation of the O3′–P bond. Comparison of the corresponding barriers and free energy changes associated with each step therefore provides insight into how Mg^2+^ coordination influences phosphodiester bond formation.

**Figure 3:**
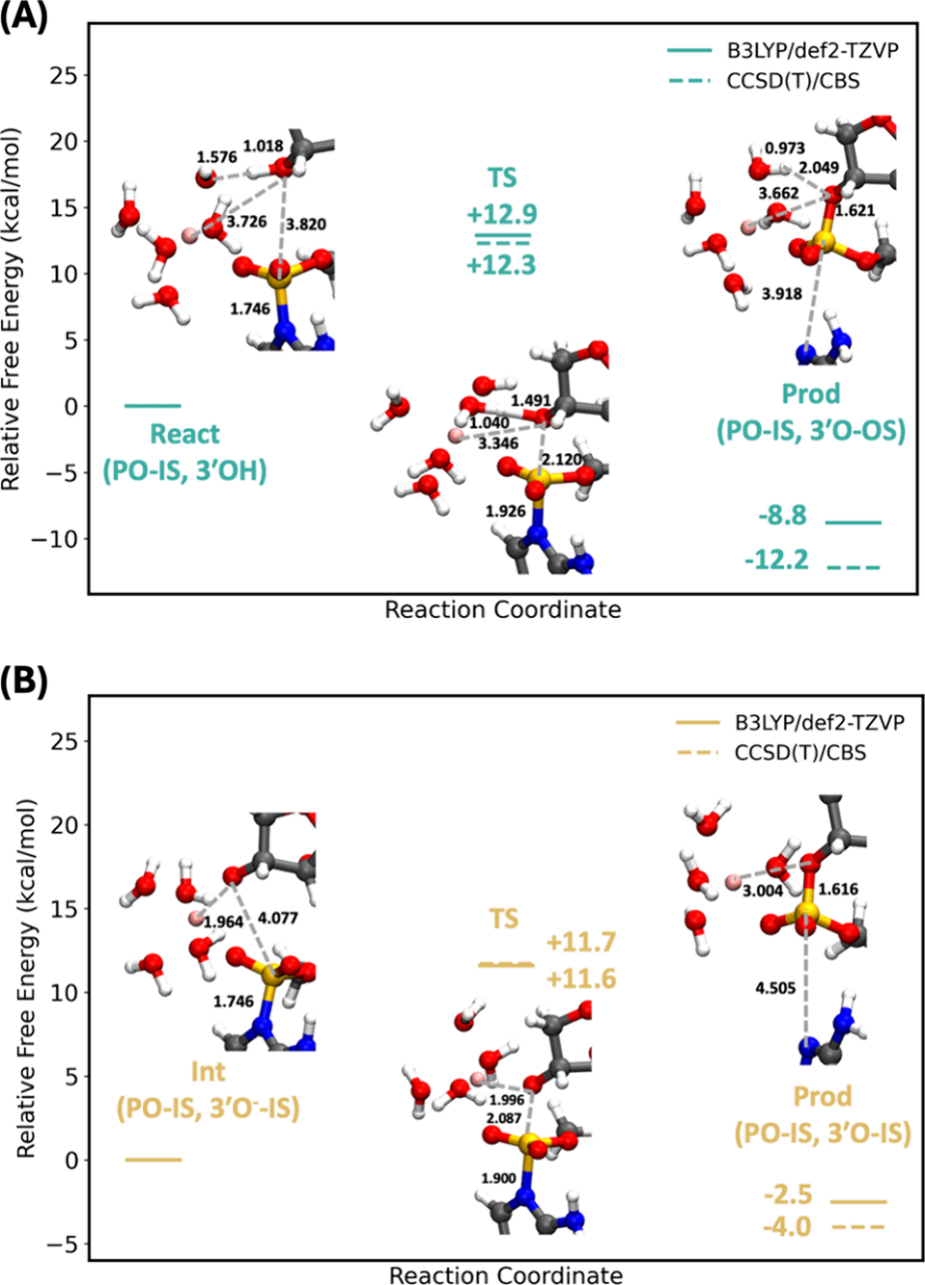
Relative free energy (kcal/mol) from the QM cluster models of the nucleophilic attack step at the B3LYP-D4/def2-TZVP (solid line) and CCSD­(T)/CBS (dashed line) level of theory with the SMD solvation model. Optimized geometry with the outer- (teal; A) and inner-sphere (beige; B) Mg^2+^–O3′ coordination is shown for each state. The interatomic distances of interest (in Å) are labeled. The relative free energies of the states are labeled next to the corresponding energy levels.

At the B3LYP-D4/def2-TZVP level of theory, the concerted, outer sphere coordinated reaction, corresponding to a direct transition from **React (PO-IS, 3′OH-OS)** to **Prod (PO-IS, 3′O-OS)** (Scheme 1B), exhibited an energy barrier of 12.9 kcal/mol. The product state was 8.8 kcal/mol lower in energy than the reactant state (Figure 3A). The nucleophilic attack step of the stepwise, inner-sphere mechanism, corresponding to the transition from **Int (PO-IS, 3′O** ^**–**^ **-IS)** to **Prod (PO-IS, 3′O-IS)**, exhibited a barrier of 11.6 kcal/mol (Figure 3B). However, the energy of the final product state was only 2.5 kcal/mol lower than that of the reactant state. Overall, DFT calculations predict that the nucleophilic attack step with inner-sphere Mg^2+^–O3′ coordination is 1.2 kcal/mol lower in barrier height than the outer-sphere pathway, but the product state is 6.3 kcal/mol higher in free energy relative to the concerted outer-sphere reaction.

The energetics calculated at the DLPNO-CCSD­(T)/CBS level of theory show similar results, with the difference in barrier reduced to 0.6 kcal/mol while the difference between reactant and product states is increased to 8.2 kcal/mol ( and Figure 3A and 3B). These findings are consistent with the ASMD results indicating a similar relative barrier height for the nucleophilic attack step with inner- and outer-sphere Mg^2+^–O3′ coordination. For the inner-sphere nucleophilic attack, the QM cluster model predicted a product state (**Prod (PO-IS, 3′O-IS)**) with 2.5 and 4.0 kcal/mol lower free energy compared to the reactant with the DFT and CC level of theory, respectively. The product geometry in the QM cluster model has a longer Mg^2+^–O3′ distance (around 3 Å; Figure 3B) compared to that at the end of the ASMD trajectory (around 2.5 Å; ). The slightly exergonic product state in the QM cluster model, together with the longer Mg^2+^–O3′ distance relative to the ASMD end point, suggests that the compact inner-sphere product geometry is not favored, likely because it requires the formation of a strained four-membered ring. This is supported by the monotonic decrease in the potential of mean force as the Mg^2+^–O3′ distance increases in the product state, indicating that the outer-sphere product geometry is the only stable minimum along this coordinate (Figure S11). Overall, the QM cluster models provide a validation of the relative energetics corresponding to the nucleophilic attack step during nonenzymatic primer extension.

### The High Barrier to Forming the Inner-Sphere Deprotonated Intermediate Favors a Concerted Outer-Sphere Reaction Pathway

While ASMD quantifies a single transition path, allowing the collective variables to vary independently exposes hidden variations along the reaction pathway. To obtain a free energy landscape for the reaction steps and their coupling to the binding mode changes with exhaustive sampling, two-dimensional (2D) QM/MM umbrella sampling was performed (Figure 4). The population histogram of each free energy landscape is used to confirm that the sampling covers the paths of interest (Figure S12). The minimum free energy paths are identified with the string method to obtain the energetics of the pathways of interest (Figure S13). The resultant energy landscapes identify three stable states: **React (PO-IS, 3′OH-OS)**, **Int (PO-IS, 3′O** ^**–**^ **-IS)**, and **Prod (PO-IS, 3′O-OS)**. The product state with inner-sphere coordination to O3′ is stable in the free energy landscape between the proton transfer and nucleophilic attack coordinate but is unstable with respect to the Mg^2+^–O3′ distance due to the strained 4-membered ring (Figure 4B,D).

**Figure 4:**
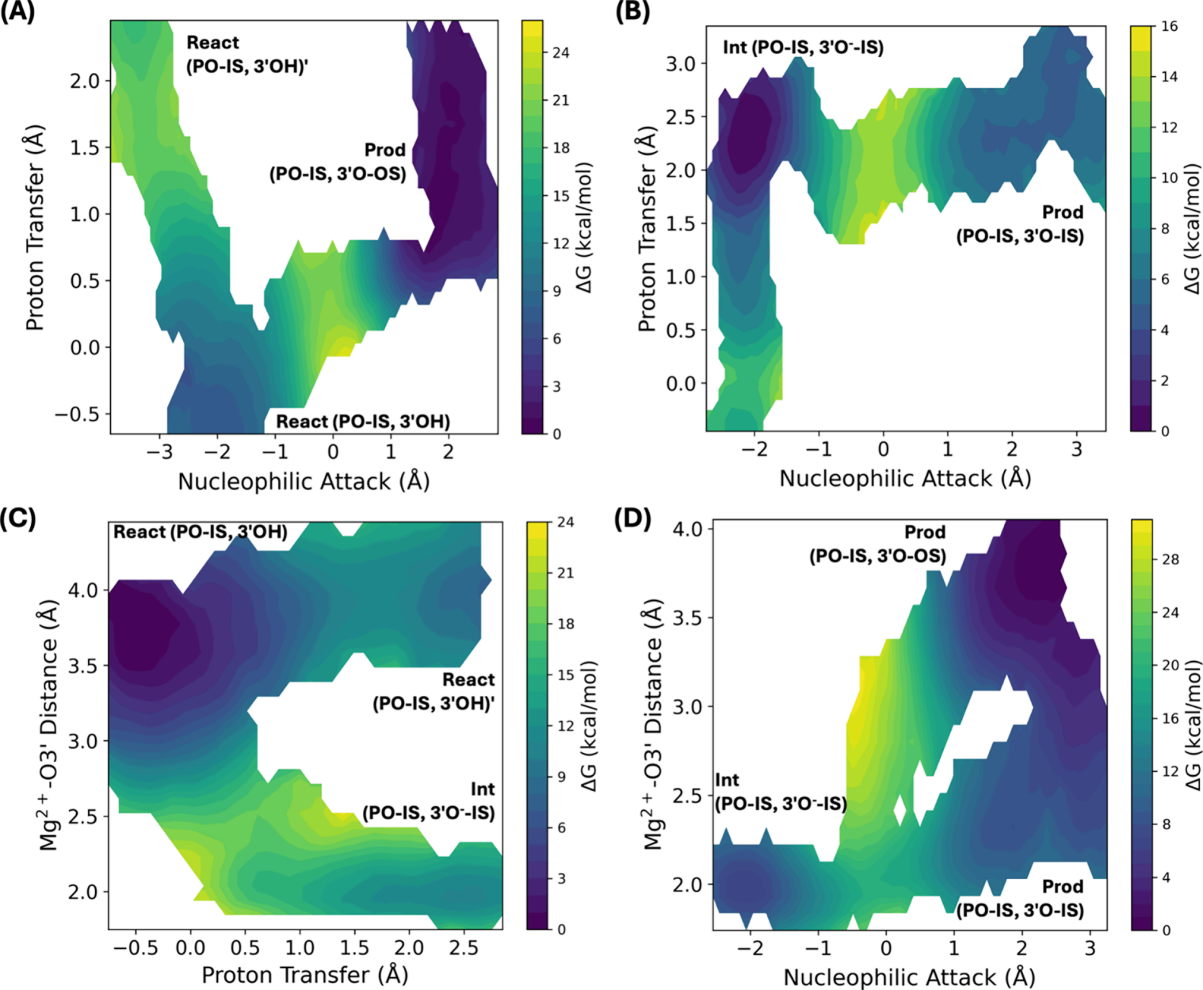
The two-dimensional free energy landscapes of collective variables of interest in nonenzymatic primer extension. Each landscape couples two dimensions of the three-dimensional free energy landscape of proton transfer, nucleophilic attack, and Mg^2+^–O3′ distance using umbrella sampling at the QM­(r^2^SCAN-3c)/MM­(OL3+TIP4P-Ew+m12–6–4) level of theory. The free energy landscape between the proton transfer and nucleophilic attack coordinate is determined with the outer- (A) and inner-sphere (B) Mg^2+^–O3′ coordination. The free energy landscape is evaluated between the Mg^2+^–O3′ distance and (C) the proton transfer coordinate before the nucleophilic attack and (D) the nucleophilic attack coordinate after the proton transfer. The states are labeled according to Scheme 1B.

Among the three stable states, two potential pathways can be identified: a pathway in which outer-sphere coordination to O3′ is continuously maintained and a pathway in which the metal ion changes from outer to inner and then back to outer-sphere O3′ coordination. In the first pathway, the proton transfer and nucleophilic attack are concerted and there is no change in Mg^2+^–O3′ coordination. This reaction is predicted to be exergonic, with a barrier height of 14 kcal/mol and a free energy change of ca. −6 kcal/mol (Figures 4A and 5). In contrast, the second pathway represents a two-step mechanism where proton transfer and nucleophilic attack occur consecutively. In the first step, Mg^2+^ changes its binding mode with O3′ from the outer- to inner-sphere coordination (Figure 4C) and the 3′OH is deprotonated. In the second step, nucleophilic attack occurs and the metal ion reverts to outer-sphere coordination (Figure 4D).

**Figure 5:**
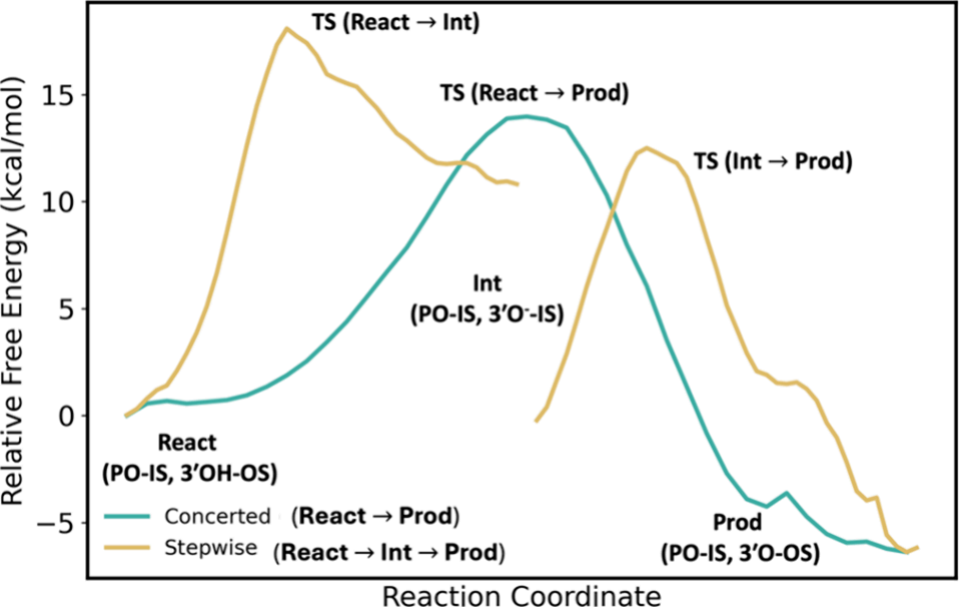
One-dimensional free energy profile for the concerted (teal) and stepwise (beige) mechanism. The minimum free energy paths are identified with the string method on the 2D QM/MM free energy landscapes. The states are labeled according to Scheme 1B. States **React (PO-IS, 3′OH-OS)** and **Prod (PO-IS, 3′O-OS)** are aligned to the same free energy between the two mechanisms.

The first step is predicted to be an endergonic process with a barrier of ca. 18 kcal/mol and a free energy change of 11 kcal/mol. The second step is a downhill step with a barrier of ca. 13 kcal/mol and a free energy change of −6 kcal/mol. Overall, this leads to a barrier of 24 kcal/mol and a free energy change of 5 kcal/mol (Figure 5). The product state of the first step (**Int (PO-IS, 3′O** ^**–**^ **-IS)**) is likely a nascent state that can be further relaxed. Together, these results suggest that the concerted mechanism with continuous outer-sphere coordination to O3′ is the most favorable pathway of the mechanisms that we have considered.

### The 2AI Bridging Group Facilitates the Unbinding of Mg^2+^ from the Pro-*S*_P_ Oxygen in the Product State

At the end of the reaction as modeled with the QM/MM scheme, the Mg^2+^ ion is inner sphere coordinated to the pro-*S*_P_ oxygen and outer sphere coordinated O3′. Relaxation of this relatively high-energy product state can then occur as the Mg^2+^ ion dissociates from the RNA and returns to bulk solution. We have modeled the initial steps in this process, and find that the catalytic Mg^2+^ can change its coordination modes to the pro-*S*_P_ oxygen and N3 of the 2AI group of the released activated mononucleotide. In principle, Mg^2+^ can coordinate to the pro-*S*_P_ oxygen, to N3, to both simultaneously, or can become solvent-coordinated without any direct interaction to either site. We refer to these states as **Prod (PO-IS)**, **Prod (N-IS)**, and **Prod (PO-IS, N-IS)**, and **Prod (PO-OS**/**N-OS)**. The solvent-coordinated state is modeled with only one outer-sphere coordination to either the pro-*S*_P_ oxygen or the N3 atom. The RESP derived charges of the activated mononucleotide are shown in Table S1.

To understand the favorable binding mode to the product and quantify their relative energetics, classical umbrella sampling was performed along the distance between Mg^2+^ and the ligand starting from the equilibrated snapshots (Sim4 to Sim6 in ). Relative to a state in which the Mg^2+^ is inner sphere coordinated to both the pro-*S*_P_ oxygen and the imidazole nitrogen (**Prod (PO-IS, N-IS)**), we examined the transition to the two states in which coordination to one of the ligands has changed to outer sphere (i.e., **Prod (PO-IS, N-OS)** and **Prod (PO-OS, N-IS)**) (Figure 6A). Based on the umbrella sampling simulations, the change from outer- to inner-sphere coordination to the imidazole N involves a barrier of 9.4 kcal/mol to reach the final state, which is 1.7 kcal/mol lower in free energy. From this bidentate state, Mg^2+^ can unbind from the pro-*S*_P_ oxygen to reach the state **Prod (PO-OS, N-IS)** with a barrier of 10.8 kcal/mol and a free energy change of −4.6 kcal/mol. Overall, the shift of the inner-sphere coordination from the pro-*S*_P_ oxygen to the imidazole nitrogen is downhill by 6.3 kcal/mol with an overall barrier of 9.4 kcal/mol. Thus, starting from the doubly inner-sphere coordinated geometry, the unbinding of Mg^2+^ from the pro-*S*_P_ oxygen is thermodynamically favorable. In contrast, Mg^2+^ prefers to remain inner sphere coordinated to the imidazole N atom, with unbinding being uphill by 3.1 kcal/mol (Figure 6B). This suggests that the 2AI bridging group facilitates the unbinding of Mg^2+^ to the pro-*S*_P_ oxygen without forming a stable bidentate complex that might hinder the dissociation of the activated mononucleotide that is the leaving group of the primer extension reaction. This preference may result in part from an entropic contribution of the highly flexible 2AI geometry in the product state. Also, the binding of Mg^2+^ may stabilize the negative charge that develops on the N atom of the departing 2AI group. The fact that the 2AI group can access regions far from the phosphate group in the product state may facilitate the dissociation of the activated mononucleotide (Figure S14).

**Figure 6:**
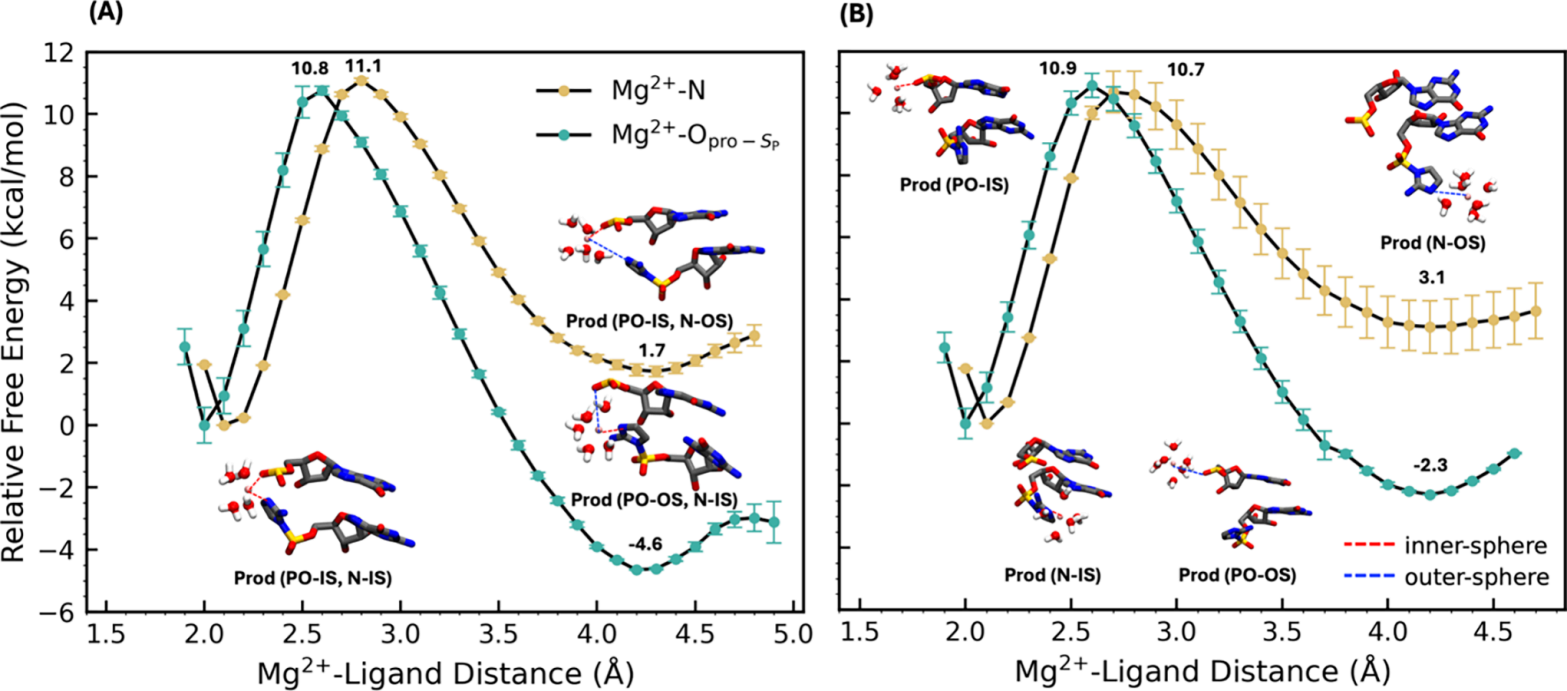
Potential of mean force as a function of the distance between Mg^2+^and the pro-*S*_P_ oxygen (teal) and the unlinked N atom (beige) in the 2AI bridging group using classical umbrella sampling. (A) The initial state **Prod (PO-IS, N-OS)** corresponds to the inner- and outer-sphere coordination of Mg^2+^ to the pro-*S*_P_ oxygen and the N atom, respectively. As the Mg^2+^ coordinates to the N atom, the system becomes the state **Prod (PO-IS, N-IS)** in which the Mg^2+^ is inner sphere coordinated to both the pro-*S*_P_ oxygen and the N atom. Mg^2+^ can then unbind from the pro-*S*_P_ oxygen and transition into the state **Prod (PO-OS, N-IS)**. These two steps are both energetically favorable and lower the free energy by 6.3 kcal/mol in total. (B) The state **Prod (PO-IS, N-OS)** and **Prod (PO-OS, N-IS)** can further undergo structural relaxations, which are denoted as **Prod (PO-IS)** and **Prod (N-IS)**, respectively. Mg^2+^ prefers to unbind from the pro-*S*_P_ oxygen in the **Prod (PO-IS)** state, which is energetically downhill by 2.3 kcal/mol, while it prefers to stay bound to the N atom in the **Prod (N-IS)** state, with the unbinding to the N atom energetically uphill by 3.1 kcal/mol. State labels are as in Scheme 1C. Beige: Mg^2+^–N distance. Teal: Mg^2+^–O­(pro-*S*_P_) distance. Error bars are the standard error of the mean from three replicates. The states **Prod (PO-IS)** and **Prod (N-IS)** are aligned to zero arbitrarily and not directly comparable in (B). Representative structures corresponding to the minimum energy are shown. The hydrogen atoms are omitted for clarity except in water.

## Discussion

The mechanism of divalent cation catalysis in nonenzymatic primer extension has been the subject of much speculation. Structural and mechanistic studies have suggested several catalytic roles for divalent cations in RNA catalysis, including stabilizing negative charge, nucleophile activation, and transition state stabilization. A previous simulation study provided support for these mechanistic explanations and demonstrated that direct, inner-sphere coordination to the deprotonated 3′-OH structurally preorganizes the reaction center for in-line attack.^9^ However, that study did not consider the entire reaction mechanism from the initial Mg^2+^ binding event through subsequent deprotonation of 3′-OH and nucleophilic attack. In the current study, we have used a multilevel approach to expand on these results and have quantified the energetics of each possible step for multiple reaction pathways. Our results suggest that primer extension likely occurs in a single, concerted step with the catalytic Mg^2+^ remaining outer sphere coordinated to the attacking O3′.

Based on the classical MD simulation, the average O3′–P distance with Mg^2+^ bridging the protonated 3′-OH and the pro-*S*_P_ phosphate oxygen is 4.60 Å, consistent with the crystal structure (Figure 1C).^8^ The increase in the O3′–P distance from 3.7 to 4.6 Å after the binding of Mg^2+^ appears to arise from disruption of the hydrogen bonding between the pro-*S*_P_ oxygen and the 3′-OH of the primer (Figure 1B). This disruption is likely due to steric effects since the first solvation shell of Mg^2+^ prohibits the formation of a hydrogen bond. Therefore, the binding of Mg^2+^ does not make the nucleophilic attack step more favorable in terms of the O3′–P distance. Experiments have shown that the nonenzymatic primer-extension reaction is highly regioselective, resulting in the formation of products with predominantly an O3′–P linkage.^24^ As shown in the MD simulation, the binding of Mg^2+^ increases the difference between the O–P distance (and the O–P–N angle) for O3′ compared to O2′. This suggests that the Mg^2+^-induced structural changes favor geometries associated with formation of O3′–P linkages over those associated with O2′–P linkages.

Our study confirms that Mg^2+^ binding to the pro-*S*_P_ oxygen promotes deprotonation of the 3′-OH. The bound Mg^2+^ lowers the p*K*_a_ of the 3′-OH group by 3.2 units according to thermodynamic integration. The p*K*_a_ of the cis-diol in nucleosides has been determined to be around 12–13 in the absence of Mg^2+^ by NMR. Therefore, the binding of Mg^2+^ in the primer extension reaction center may decrease the p*K*_a_ of the primer 3′-OH to about 9–10. This range is consistent with the results of pH-rate profiling, which show that deprotonation of some group with a p*K*_a_ of 9.1 is necessary for the reaction to occur.^12–16^ Our results show that the binding of Mg^2+^ to the pro-*S*_P_ oxygen of the reactive phosphate group can account for this degree of lowering of the 3′-OH p*K*_a_, thereby making the proton transfer step of primer extension more thermodynamically favorable. At least two factors may contribute to the Mg^2+^-induced decrease in the 3′-OH p*K*_a_. First, the positively charged Mg^2+^ should stabilize the deprotonated 3′-OH group electrostatically. Second, loss of the hydrogen bond between the 3′-OH and the nonbridging phosphate oxygen should favor deprotonation.

What is the most likely pathway for deprotonation of the 3′-OH group? Multiple potential deprotonation pathways of the 3′-OH group exist. In enzymatic reactions such as polymerization, the proton can be transferred from the primer 3′-OH to the bulk solvent via substrates (e.g., the 2′-OH group or phosphate oxygens), adjacent protein residues, or hydroxide ions bound to metal cofactors, or directly to solvent hydroxide ions acting as a general base.^25^ A previous structure–activity study of nonenzymatic RNA primer extension has shown that a 2′-fluoro-substituted attacking nucleotide at the 3′-end of the primer increases the reaction rate by approximately 2-fold instead of halting the reaction.^16^ Since this substitution does not significantly change the structure of the reaction center, it is unlikely that the 2′-OH group mediates the proton transfer in the canonical reaction system. Since the O3′–P distance is increased when Mg^2+^ is bound, the proton is unlikely to be transferred to the phosphate oxygen (). We suggest that a metal-bound hydroxide is the most likely proton acceptor since it can bridge the 3′-OH group with hydrogen bonding and the metal center. Therefore, in our simulations, we used a Mg^2+^-bound hydroxide ion as the general base when modeling the reaction.

To determine the optimal starting state for modeling the phosphodiester bond formation, the nature of the Mg^2+^–O3′ coordination after Mg^2+^ binding to the pro-*S*_P_ oxygen must be identified. Classical umbrella sampling shows that the outer-sphere coordination to the 3′-OH is more favorable thermodynamically than inner-sphere coordination in the absence of a Mg^2+^-bound hydroxide (Figure 2B). However, this does not categorically eliminate the involvement of the inner sphere coordinated state in the reaction mechanism, since it may be possible to transiently populate that state despite its thermodynamic disadvantage. Therefore, we have investigated both the inner- and outer-sphere coordination of Mg^2+^ to the 3′-OH as potential intermediates in the reaction mechanism.

We begin by considering the state in which the Mg^2+^ is inner sphere coordinated to the pro-*S*_P_ oxygen of the reactive phosphate group, and outer sphere coordinated to the 3′-OH. This is a reasonable starting point for modeling the reaction, since it is the lowest energy mode for Mg^2+^ binding in the reaction center. Since the phosphoryl transfer reaction requires a strong nucleophile, we assume that the deprotonation step either precedes or occurs simultaneously with the nucleophilic attack, corresponding to a stepwise and concerted mechanism, respectively. The metal-bound hydroxide can deprotonate the 3′-OH group of the attacking nucleotide with Mg^2+^ interacting either directly with the 3′-OH group or via outer-sphere coordination. The deprotonation step can also be coupled to a binding mode change from outer- to inner-sphere coordination of Mg^2+^ to O3′. In the latter case, when Mg^2+^ is pulled from outer- to inner-sphere coordination in the ASMD trajectory (), the proton transfer step happens spontaneously. This is because, as shown in the 2D free energy landscape between the proton transfer coordinate and the Mg^2+^–O3′ distance, the protonated inner-sphere state lies in a high-energy region (Figure 4A).

When steered along the proton transfer coordinate, the 3′-O^–^ is immediately reprotonated by an adjacent Mg^2+^-bound water molecule, resulting only in a net displacement of hydroxide. This is contrary to the stable state observed in classical umbrella sampling, where such a deprotonation-reprotonation step is not possible without a reactive force field or quantum chemical treatment. The displaced-hydroxide state exhibits a smaller average O3′–P–N angle compared to the starting state, which is unfavorable for the nucleophilic attack. Given that the barrier for hydroxide displacement is comparable to that of the concerted proton transfer and nucleophilic attack with the outer-sphere Mg^2+^–O3′ coordination, this step likely represents an off-pathway rearrangement of the reaction center that competes with the productive pathway. This also indicates that the relative position of the metal-bound hydroxide ion can affect the reaction rate. In the last case we investigated, we steered the proton transfer at the same time as the Mg^2+^–O3′ binding mode change from the outer- to the inner-sphere coordination. The 2D free energy landscape confirmed that inner-sphere coordination to 3′-O^–^ is a metastable state that could potentially be followed by the nucleophilic attack step. We did not further consider inner-sphere coordination to the protonated 3′-OH because that state is unstable, and we also eliminated outer-sphere coordination to deprotonated 3′-O^–^ which is an off-pathway species.

The nucleophilic attack step can therefore start with the Mg^2+^ either outer-sphere coordinated to the 3′-OH, via a concerted proton transfer and nucleophilic attack step, or from the transient state in which the Mg^2+^ is inner-sphere coordinated to the already deprotonated 3′-O^–^. However, the free energy landscape shows that a product state with an inner-sphere Mg^2+^–O3′ coordination is not stable because of the strained 4-membered ring Mg^2+^–O3′–P–O­(pro-*S*_P_). Therefore, as the nucleophilic attack step proceeds, the Mg^2+^ must move toward outer-sphere coordination with O3′ after passing the transition state. As expected, this binding mode change relaxes the strained four-membered coordination among the pro-*S*_P_ oxygen, O3′, P, and Mg^2+^. The barrier for this pathway obtained from QM/MM umbrella sampling (13 kcal/mol) is similar to that for concerted proton transfer and nucleophilic attack step with the outer-sphere Mg^2+^–O3′ coordination (14 kcal/mol). Thus, the barrier for the nucleophilic attack step is not sensitive to the mode of Mg^2+^–O3′ coordination. This is also manifested in the 2D free energy landscape coupling the nucleophilic attack and the Mg^2+^–O3′ distance, which is relatively flat along the Mg^2+^–O3′ distance (Figure 4D). Nevertheless, nucleophilic attack starting from a state where the Mg^2+^ is inner-sphere coordinated to 3′-O^–^ appears to be unfavorable because that starting state is a high-energy metastable state.

With the energetics of the proton transfer and nucleophilic attack step thus elucidated, we propose the following overall reaction mechanism:

1) Mg^2+^ binds to the pro-*S*_P_ oxygen of the reactive phosphate group from the bulk solution and remains outer sphere coordinated to O3′ (**Pre (PO-OS)** *→* **Pre (PO-IS, 3′OH-OS)**);
2) One of the Mg^2+^-bound water molecules is deprotonated to form a metal-bound hydroxide ion (**Pre (PO-IS, 3′OH-OS)** *→* **React (PO-IS, 3′OH-OS)**);
3) Mg^2+^ remains in outer-sphere coordination with the protonated 3′-OH group;
4) The 3′-OH group is deprotonated by a metal-bound hydroxide ion as the nucleophilic attack from O3′ to P happens (**React (PO-IS, 3′OH-OS)** *→* **Prod (PO-IS, 3′O-OS)**);
5) The system further relaxes from the nascent product state.

Based on this mechanism, our simulations predict that the nonenzymatic primer-extension reaction is an exergonic reaction with a free energy change of ca. −3 kcal/mol and an overall barrier of ca. 23 kcal/mol. The barrier includes the free energy change for Mg^2+^ to go from outer- to inner-sphere coordination to the pro-*S*_P_ oxygen (∼5 kcal/mol), the free energy change for a metal-bound water to deprotonate and form a hydroxide ion (∼4 kcal/mol), and the reaction barrier for the concerted proton transfer and nucleophilic attack step (∼14 kcal/mol). The calculated barrier is consistent with a reaction barrier of 20 kcal/mol, corresponding to the experimentally observed reaction rate in a similar RNA primer/template/substrate complex.^26^ Our proposed mechanism is also consistent with experimental kinetic studies that suggest that the nucleophilic attack step is the rate-limiting step of the reaction.^16^ The structural relaxations as observed in MD simulations are expected to further stabilize the product state, although the corresponding free energy change has not been quantified in the present analysis.

The catalytic role of Mg^2+^ identified in this study is consistent with previous mechanistic studies of ribozymes, such as the Varkud satellite ribozyme,^27^ the hammerhead ribozyme,^28,29^ and the hepatitis delta virus ribozyme,^30^ in which Mg^2+^ contributes to structural organization and/or p*K*_a_ tuning. In the current nonenzymatic system, the catalytic Mg^2+^ disrupts the hydrogen bonding to the reactive phosphate group and lowers the p*K*_a_ of the nucleophilic 3′-OH group to facilitate its activation, paralleling catalytic strategies described for RNA-cleaving enzymes.^31,32^

Compared with highly evolved enzymes^33,34^ and designed nanozymes^35^ for phosphate chemistry, which often employ a more tightly organized reaction center and one or more precisely placed catalytic metal ions, the thermodynamically costly Mg^2+^ binding to the pro-*S*_P_ oxygen and the preferred outer-sphere coordination to O3′ observed here may reflect limited preorganization of the reaction center.^36–38^ In ribozymes or protein enzymes, preorganized active sites can stabilize inner-sphere metal–ligand coordination modes that are less favorable in a flexible nonenzymatic reaction center. From an evolutionary perspective, one possible interpretation is that, as catalytic systems acquired greater structural organization in ribozymes and proteins, tighter and more selective metal-ion binding became increasingly accessible for phosphoryl-transfer reactions.^39^

Based on our computational modeling of the reaction, we propose the following design principles to enhance the rate of nonenzymatic RNA primer extension. The binding of Mg^2+^ to the pro-*S*_P_ oxygen contributes around a fifth of the overall barrier. Thus, in addition to continued exploration of the activating group design, it would be beneficial to identify prebiotically plausible molecules that can more favorably facilitate the binding of the catalytic Mg^2+^ to the reaction center. For example, a short peptide that could both coordinate the metal and bind to RNA might be helpful.

Such a peptide or other molecule could also help to resolve the incompatibility between the high concentrations of divalent metal ions needed for template-directed primer extension and the fatty acid membranes used in some models of primordial cells. For a system to replicate and undergo Darwinian evolution, it is necessary to compartmentalize genetic replicators so that they can pass on advantageous mutations to daughter cells.^40^ Fatty acid membranes are promising in this regard because of their permeability, stability, and ability to grow and divide.^41^ High concentrations of metal ions, however, precipitate fatty acids and disrupt or destroy the membranes. One way to resolve this incompatibility is to chelate the Mg^2+^ in a manner that protects the fatty acid membrane without disrupting the interactions that are essential for Mg^2+^ catalysis of primer extension.^7^ The atomistic simulations presented here show that two coordination sites of Mg^2+^ participate in the preferred reaction pathway, suggesting that chelators engaging no more than four coordination sites may be promising candidates for future testing.

Lastly, we note that the stepwise pathway involving deprotonation and nucleophilic attack with inner-sphere Mg^2+^–O3′ coordination could become viable if that initial state was stabilized. Such a change of mechanism could potentially occur with alternative metal ions such as Mn^2+^ or Fe^2+^, or if the metal ion is chelated with a small molecule or peptide that stabilizes a different metal binding mode.

## Conclusions

We have used multiscale QM/MM methods to investigate the mechanism by which Mg^2+^ catalyzes nonenzymatic RNA primer extension. Our results suggest that the binding of Mg^2+^ to the pro-*S*_P_ oxygen of the reactive phosphate group of the 2AI-bridged dinucleotide substrate is energetically uphill. However, the presence of the bound Mg^2+^ lowers the p*K*_a_ of the 3′-OH group of the terminal primer nucleotide by around 3.2 units, setting the stage for nucleophilic attack on the reactive phosphate. Two-dimensional free energy simulations identified a previously uncharacterized energy landscape for nonenzymatic primer extension in which proton transfer and nucleophilic attack proceed most favorably through a concerted process, with Mg^2+^ remaining inner-sphere coordinated to the pro-*S*_P_ oxygen and outer-sphere coordinated to O3′. The reaction is predicted to be exergonic with a barrier of ca. 23 kcal/mol, consistent with experimental results. We expect that this computational framework can be extended in future studies to investigate how other catalytic divalent metal ions, such as Mn^2+^ and Fe^2+^, can modulate the landscape of this reaction, and potentially increase the rate and extent of template copying in a prebiotically plausible fashion.

## Methods

### System Setup

Classical molecular dynamics simulations were carried out in the AMBER software using the single-GPU version of PMEMD.^42^ The system was built with AmberTools.^43^ The standard AMBER OL3 force field (χOL3) was used for the canonical nucleotides as well as the nonstandard nucleic-acid-like residues, including 2AI-bridged dinucleotides and 3′-OH-deprotonated nucleotides.^44^ The force field parameters involving the 2AI bridging group were taken from the *ff19SB* force field^45^ and the parameters for phosphorylated amino acids.^46^ Improper dihedral angle parameters were not included for the bridging group. The charges of the nonstandard residues were derived using the restrained electrostatic potential (RESP) charges^47^ at the HF/6–31G* ^48–50^ level of theory with the R.E.D. Server.^51^ The initial coordinates of the RNA duplex were taken from a previous study, in which the RNA duplex with bound 2AI-bridged dinucleotide was generated by aligning to the crystal structure (PDB: 6C8E).^9^ The system was solvated in a TIP4P-Ew water with the dimensions of about 78 Å.^52^ To model the experimental MgCl_2_ concentration within the 100 mM range used for catalysis, we used a MgCl_2_ concentration of around 0.15 M while maintaining overall charge neutrality.^53^ In the pre-reaction-state simulations, 43 Mg^2+^ ions were modeled with the fine-tuned divalent cation parameters for nucleic acids^54,55^ and neutralized with 66 Cl^–^ ions. The initial coordinates of ions were taken from the previous study, in which the ions had been placed with the command *autoionize* in Visual Molecular Dynamics (VMD).^56^ In the product-state simulations, the ions were added in solution with the tLeap command *addIonsRand*. The number of ions was adjusted based on the overall charge for simulating the deprotonated intermediate and product. The systems were equilibrated for at least 100 ns before structural analysis and free energy calculations to allow redistribution of solvent and ions. ParmEd was used to edit the topology files.^57^

### Classical Molecular Dynamics Protocol

AMBER was utilized as the MD engine in this study. A cutoff of 9 Å was used with the particle-mesh Ewald method for periodic boundary conditions.^58,59^ An initial minimization of the system was performed using the steepest descent method for 500 steps, followed by the conjugate gradient method for another 500 steps. The RNA duplex and the Mg^2+^ ion bound to the reactive phosphate group in the 2AI-bridged dinucleotide were restrained with a harmonic potential of 10 kcal mol^–1^ Å^–2^. This potential was then lifted for the system to further minimize for 1,000 and 1,500 steps with the steepest descent and the conjugate gradient method, respectively. After minimization, the SHAKE^60^ algorithm was switched on to allow a time step of 2 fs during the simulation. To establish the NVT ensemble, the Langevin thermostat was utilized with a collision frequency of 1 ps^–1^. The system was heated from 0 to 300 K within 2 ps with the restraining potential of 10 kcal mol^–1^ Å^–2^ on the RNA duplex and equilibrated for a total of 100 ps. An equilibration in the NPT ensemble was then carried out with the Monte Carlo barostat at 1 atm. Flat-bottom constraints with a cutoff of 3.0 Å with 100 kcal mol^–1^ Å^–2^ or harmonic constraints of 50 kcal mol^–1^ Å^–2^ on the Mg^2+^-ligand distance were applied when necessary to maintain the coordination mode of interest in the product state. VMD was used for visualization of the system and collective variable analysis of the MD trajectories. Collective variables were taken with a stride of 0.1 ns for analysis.

### Thermodynamic Integration

Thermodynamic integration was used to evaluate the p*K*_a_ shift of the 3′-OH group of the attacking nucleotide induced by the binding of Mg^2+^ to the pro-*S*_P_ oxygen of the reactive phosphate group. A time step of 1.0 fs was used with the SHAKE algorithm turned off between O3′ and the proton. The second-order softcore potential was applied to both electrostatic and van der Waals interactions with *m = n =* 2, α = 0.5, β = 1.0, and the control flag *gti_add_sc =* 5.^61,62^ For both interactions, the potential was smoothly cut off between 7 and 9 Å. To maintain charge neutrality, the proton of the 3′-OH group was mutated to a dummy atom together with a Cl^–^ ion, following the co-alchemical ion approach.^63^ To prevent this co-alchemical ion from interacting with the RNA duplex, a constraining potential with a flat bottom in the range of 30 to 42 Å was applied between the geometric center of the phosphorus atoms of the RNA duplex and the co-alchemical Cl^–^. A harmonic potential of 10 kcal mol^–1^ Å^–2^ is applied if the ion exceeds no more than 2 Å outside the range and becomes linear for larger deviations to avoid tearing up the duplex. The transformation is divided into 11 windows from λ = 0.0 to 1.0 with an increment of 0.1. Each window was equilibrated for 15 ns, and the last frame was then used as the starting structure for the next window. For each window, a production run of 5 ns was replicated three times to estimate the error. Additional windows were added at the midpoints of the initial windows and in regions where the derivatives changed sharply (λ = 0.52, 0.525, 0.53, 0.535, 0.54, 0.545, 0.575, 0.585, 0.625, and 0.775). Windows with large statistical uncertainties (λ = 0.50 for the bound state and λ = 0.80 for the unbound state) were extended to 15 ns, while windows in the region with sharp derivative changes (λ = 0.525, 0.53, 0.535, and 0.54) were extended to 30 ns. The package *alchemlyb* was utilized for data processing and free energy calculation.^64^ The change in the free energy change of this transmutation between the two states, with and without Mg^2+^ bound to the pro-*S*_P_ oxygen, was used to calculate the Mg^2+^-induced p*K*_a_ shift.

### Hybrid QM/MM MD Simulations

The QM/MM simulation was performed with the extensible interface^65^ implemented in AMBER, with ORCA^66^ as the external QM program. The system was divided into a quantum region treated with the electronic structure method, with the rest at the MM level. To balance the accuracy and computational cost, the QM region includes the sugar ring of the attacking nucleotide, the part between the two C5′ atoms in the 2AI-bridged dinucleotide, the Mg^2+^ ion, and its coordinating water molecules, totaling 52 atoms with a neutral charge. Hydrogen atoms were added at the QM/MM boundary as link atoms. The residual charges were distributed over the MM atoms bonded to the QM atoms at the QM/MM boundary. For the QM waters, the extra point charges used in the TIP4P-Ew model were stripped. This QM region was treated with the r^2^SCAN-3c method within the electrostatic embedding scheme ( for cluster-model benchmarking).^67^ QM calculations for each time step used TightSCF convergence criteria in ORCA.

Since the particle mesh Ewald method is not supported in the sander/ORCA interface, a nonperiodic droplet model was adopted for the QM/MM simulations. A water droplet with a radius of 36 Å was extracted from an equilibrated snapshot with periodic boundary conditions and used for subsequent QM/MM simulations. A solvent cap restraint potential of 1.5 kcal mol^–1^ Å^–2^ was applied when water molecules were farther than 36 Å away from the geometric center of the duplex to establish spherical boundary conditions. A cutoff of 999 Å is used so that effectively all the QM/MM and MM/MM interactions are included.^68^ Similar droplet-based QM/MM protocols have been benchmarked previously,^69^ while fully periodic treatments such as the ambient-potential composite Ewald method^70^ provide an alternative approach. Each QM/MM step integrates over 0.5 fs with the SHAKE algorithm switched off for the QM region.

### Adaptive Steered Molecular Dynamics

An equilibrated snapshot from a nonperiodic QM/MM equilibration is selected with Mg^2+^ interacting with O3′ via outer-sphere coordination. Three reaction coordinates were included in the steering, including the proton transfer coordinate (PT), nucleophilic attack coordinate (NA), and the Mg^2+^–O3′ distance. PT and NA are defined as the distance difference between the bond being broken and the bond being formed:

$$\mathrm{PT}=d_{{O3}^{\prime }-{H3}^{\prime }}-d_{{H3}^{\prime }-O_{w}}$$

$$\mathrm{NA}=d_{P-N}-d_{O3’-P}$$

Each reaction coordinate of interest is divided evenly into around 10 stages. For each stage, a moving harmonic potential of 200 kcal mol^–1^ Å^–2^ is applied to the reaction coordinate with PLUgin for MolEcular Dynamics (PLUMED).^71^ The associated pulling work is documented for each time step. A total of 10 trajectories are launched in parallel, each lasting 1 ps. The Jarzynski average of the 10 trajectories in each stage is calculated as:

$$\Delta F=-\frac{1}{\beta }\ln\left\langle e^{-\beta W}\right\rangle$$

The last frame of the trajectory closest to the Jarzynski average of the current stage is used as the starting coordinate for the next stage with a randomly assigned velocity. A snapshot equilibrated for at least 1 ps is selected to be steered along the next reaction coordinate. The end point of each ASMD simulation is equilibrated for around 5 ps to ensure that the collective variables of interest, the RMSD of the attacking nucleotide and the 2AI-bridged dinucleotide, and the electronic energy of the QM region are equilibrated.

As shown in Scheme 1B, the reaction coordinates describing the chemical reaction steps include the proton transfer coordinate for the deprotonation of the 3′-OH group and the phosphoryl transfer coordinate for the nucleophilic attack. ASMD simulations were carried out by steering the proton transfer and nucleophilic attack coordinate separately and concertedly when Mg^2+^ stays in the outer-sphere coordination. To investigate the coupling between binding mode changes and each reaction step, the Mg^2+^–O3′ distance is added as a collective variable, where the inner- and outer-sphere coordination corresponds to around 2.0 and 4.0 Å, respectively. To prevent the coordination of MM water molecules with Mg^2+^ during binding mode changes, each steering trajectory involving binding mode changes starts with the outer-sphere coordination state. Besides just steering the binding mode change, the following coupled steps are studied: i) the proton transfer step and a binding mode change from the outer- to inner-sphere coordination (**React (PO-OS, 3′OH-OS)** *→* **Int (PO-IS, 3′O** ^**–**^ **-IS)**) and ii) the nucleophilic attack step and a binding mode change from the inner- to outer-sphere coordination (**Int (PO-IS, 3′O** ^**–**^ **-IS)** *→* **Prod (PO-IS, 3′O-OS)**). During all the simulations above, the PT and NA coordinates are steered at 0.5 Å/ps, with the PT coordinate slowed to 0.12 Å/ps in the concerted pulling. The Mg^2+^–O3′ distance is steered at 0.18 Å/ps for each simulation.

### QM Cluster Models

ORCA is used for quantum chemistry calculations of the cluster models. For each energy minimum, three initial structures were extracted from the snapshots with the lowest QM polarized electronic energies in the unbiased QM/MM equilibration that are separated by at least 0.5 ps. The QM cluster models with the inner- and outer-sphere Mg^2+^–O3′ coordination are constructed by taking the QM region capped with H atoms at the QM/MM boundary (53 and 56 atoms, respectively). The geometries of the clusters are optimized at the B3LYP^72–74^ /def2-TZVP^75^ level of theory with the D4 dispersion correction.^76^ The universal solvent model based on density (SMD) is used to incorporate the solvent effects of water surrounding the cluster.^77^ The climbing image nudged elastic band (CI-NEB) method was employed to obtain the transition state guesses.^78^ In order to maintain biological relevance, the four heavy atoms at the truncation boundary are kept frozen for all optimizations, including the C1′, C4′ of the attacking nucleotide, and C5′ of each nucleotide of the 2AI-bridged dinucleotide. In the product states (**Prod (PO-IS, 3′O-IS)** and **Prod (PO-IS, 3′O-OS)**), the torsion angle C4′-C3′-O3′-P is constrained during optimization. Any spurious hydrogen bonding that appeared after initial optimization is prevented by constraining the bond length of the two atoms involved. Frequency analysis at the same theoretical level is performed to obtain the thermodynamic correction. The structural constraints led to small, nonreactive imaginary frequencies (<50*i* cm^–1^) localized on restrained atoms. DLPNO-CCSD­(T)^79^ calculations were performed on the optimized geometries at the B3LYP-D4/def2-TZVP­(SMD) level of theory with ORCA. SMD was employed to approximate the solvent effects. The energies were extrapolated to the complete basis set limit with the cc-pVTZ and cc-pVQZ basis sets.^80–82^ TightSCF and TightPNO were specified in the calculation. The thermodynamic correction from the DFT calculation was used to estimate the free energy.

### Classical Umbrella Sampling

To determine the free energy landscape along the Mg^2+^–O­(pro-*S*_P_) distance and the Mg^2+^–O3′ distance with the protonated and deprotonated 3′-OH group, umbrella sampling is performed in the range of 1.9–4.8 Å, 2.0–4.5 Å, and 1.9–4.1 Å using 30, 26, and 23 windows each equilibrated for 15 ns. A harmonic constraint of 200, 100, and 250 kcal mol^–1^ Å^–2^ is applied with NMR constraints in AMBER, respectively. In the product state, the equilibrated snapshot with Mg^2+^ inner sphere coordinated to both the unlinked N atom in the bridging group and the pro-*S*_P_ oxygen is selected. Short, biased simulations of adjacent windows are used to generate the initial snapshots close to the collective variable of each window. Umbrella sampling is performed along the Mg^2+^–N and Mg^2+^–O­(pro-*S*_P_) distance in the range of 2.0–4.8 and 1.9–4.9 Å using 29 and 31 windows. A harmonic constraint of 200 and 100 kcal mol^–1^ Å^–2^ is applied with NMR constraints in AMBER, respectively. For the relaxed product states, the unbinding is sampled along the Mg^2+^–N and Mg^2+^–O­(pro-*S*_P_) distance in the range of 2.0–4.7 Å and 1.9–4.6 Å, both with 28 windows and a harmonic constraint of 100 and 200 kcal mol^–1^ Å^–2^, respectively. Umbrella sampling windows for the product states are pre-equilibrated for 15 ns, followed by production runs each of 15 ns. The PMF is obtained from biased MD simulations with the Weighted Histogram Analysis Method (WHAM).^83^ Three replicates are used to estimate the error of the potential of mean force.

### QM/MM Umbrella Sampling

To determine whether the proton transfer and nucleophilic attack step happen in a stepwise or concerted manner, 2D QM/MM umbrella sampling is carried out along these two reaction coordinates with both the inner- and outer-sphere coordination between Mg^2+^ and O3′. In addition, to quantify the dependence of the reaction steps on the metal ion binding mode, the free energy landscape of proton transfer with the protonated 3′-OH group and nucleophilic attack with the deprotonated 3′-OH group is also calculated with varying Mg^2+^–O3′ distances. A harmonic constraint between 80 and 160 kcal mol^–1^ Å^–2^ is used in the QM/MM umbrella sampling along the reaction coordinates with PLUMED. The starting coordinates for each window were taken from the ASMD and equilibration simulations and equilibrated for 1.5 ps. The minimum free energy paths were identified with the string method.^84^ For each path, at most 2000 steps were performed with a step size of 0.1 and 40 nodes in total.

## Supplementary Material





## Notes

Charges, force field parameters, topology files, coordinate files, and input files for classical and QM/MM simulations; geometries of QM cluster models; animation of trajectories from adaptive steered molecular dynamics of the concerted and stepwise mechanism (ZIP)
Details of the RESP charge-fitting protocol and charges for the 2AI-bridged dinucleotide, deprotonated nucleotide, and activated mononucleotide; summary of classical simulations performed; energetics from QM/MM adaptive steered molecular dynamics; energetics from quantum cluster models; snapshot of the 2AI-bridged dinucleotide parametrized with the GAFF force field and AM1-BCC charges; distance distributions between hydroxyl protons and phosphate oxygens in the pre-reaction geometry; derivatives of the Hamiltonian with respect to the alchemical parameter for each thermodynamic-integration window; minimum Mg^2+^–O­(pro-*S*_P_) distance in classical simulations; potentials of mean force obtained from adaptive steered molecular dynamics; population histograms from 2D QM/MM umbrella sampling; minimum free-energy paths on the 2D free-energy landscapes; and distance distributions between the bridging group and phosphate in the product state (PDF)
