# Supplementary material for: Mg2+ Catalyzes Nonenzymatic RNA Primer Extension through a Concerted Outer-Sphere Mechanism
Source: J Am Chem Soc. 2026 Jun 8;148(23):24379–94. doi: 10.1021/jacs.6c06167 (PMC13281521; doi:10.1021/jacs.6c06167)
Supplement: Supplementary file 2 [file ja6c06167_si_002.pdf]

Supporting Information for

Mg^{2+} Catalyzes Nonenzymatic RNA Primer Extension through a Concerted Outer-Sphere Mechanism

Ruohe Wang[1,2,3], Collin Nisler[1,3], Qiang Cui[4,5,6], Laura Gagliardi*[1,2,7] and Jack W. Szostak*[1,3]

[1]Department of Chemistry, University of Chicago, Chicago, Illinois 60637, United States

[2]Chicago Center for Theoretical Chemistry, University of Chicago, Chicago, Illinois 60637, United States

[3]Howard Hughes Medical Institute, University of Chicago, Chicago, Illinois 60637, United States

[4]Department of Chemistry, Boston University, Boston, Massachusetts 02215, United States

[5]Department of Physics, Boston University, Boston, Massachusetts 02215, United States

[6]Department of Biomedical Engineering, Boston University, Boston, Massachusetts 02215, United States

[7]Pritzker School of Molecular Engineering, University of Chicago, Chicago, Illinois 60637, United States

Email: lgagliardi@uchicago.edu, jwszostak@uchicago.edu

Table of Contents

Table S1. RESP Charges for 2AI-Bridged Diguanosine **(A)**, Deprotonated Guanosine **(B)**, and Activated Guanosine **(C)** S2-S3

Table S2. Summary of Classical Simulations Performed in This Study S4

Table S3. Energetics from QM/MM Adaptive Steered Molecular Dynamics S5

Table S4. Energetics from QM Cluster Models S6

Figure S1. Snapshot of 2AI-bridged dinucleotide and the attacking nucleotide with the 2AI-bridged dinucleotide parameterized using the GAFF2 force field and the AM1-BCC charges. S7

Figure S2. The distance distributions between the proton of the 2′- and 3′-OH group and the pro-S_P and pro-R_P oxygen with and without Mg^{2+} bound to the pro-S_P oxygen S8

Figure S3. Average of the derivatives of Hamiltonian with respect to alchemical parameter in each window. S9

Figure S4. The minimum distance between Mg^{2+} and the pro-S_P oxygen (in Å) in classical equilibrium simulations S10

Figure S5. Potential of mean force obtained with adaptive steered molecular dynamics for the pathway **React (PO-IS, 3′OH-OS)** to **React (PO-IS, 3′OH-IS)** S11

Figure S6. Potential of mean force obtained with adaptive steered molecular dynamics for the pathway **React (PO-IS, 3′OH-OS)** to **Int (PO-IS, 3′O$^-$-OS)** S12

Figure S7. Potential of mean force obtained with adaptive steered molecular dynamics for the pathway **React (PO-IS, 3′OH-OS)** to **Int (PO-IS, 3′O$^-$-IS)** S13

Figure S8. Potential of mean force obtained with adaptive steered molecular dynamics for the pathway **React (PO-IS, 3′OH-OS)** to **Prod (PO-IS, 3′O-OS)** S13

Figure S9. Potential of mean force obtained with adaptive steered molecular dynamics for the pathway **Int (PO-IS, 3′O$^-$-IS)** to **Prod (PO-IS, 3′O-IS)** S14

Figure S10. Potential of mean force obtained with adaptive steered molecular dynamics for the pathway **Prod (PO-IS, 3′O-OS)** to **Int (PO-IS, 3′O$^-$-IS)** S14

Figure S11. Potential of mean force obtained with adaptive steered molecular dynamics for the pathway **Prod (PO-IS, 3′O-OS)** to **Prod (PO-IS, 3′O-IS)** S15

Figure S12. Population histogram of the two-dimensional QM/MM umbrella sampling S16

Figure S13. The minimum free energy paths identified with the string method on the two-dimensional free energy landscapes from QM/MM umbrella sampling. S17

Figure S14. The distance (in Å) between the P atom in the newly formed phosphodiester bond and the unlinked N atom S18

RESP Charge Fitting Protocol

The RESP charge is derived for the 2AI-bridged dinucleotide and the deprotonated nucleotide at the HF/6-31G* level of theory with Gaussian09[1] as described in Cornell et al. In the fitting procedure, four nucleosides (A, U, G, C) are used to impose the intermolecular constraint that the sugar atoms except C1′ and H1′ share the same charges among the nucleotides. Four and six orientations are generated by the rigid-body reorientation algorithm and used for the linker and the nucleosides, respectively.

Deprotonated Dinucleotide. The dihedral angle of the 2′-OH group is constrained to 290 degrees. The charges of O3′, P, O1P, O2P, and O5′ in the (*g,g)*-dimethyl phosphate and the 5′-OH group in the deprotonated nucleoside are kept the same as in the canonical nucleoside. The intermolecular constraint is imposed that the sum of the charges of the 5′-OH group and the methyl group connected to O5′ in the (*g,g)*-dimethyl phosphate is neutral.

2AI-Bridged Dinucleotide. The optimized structure of the four canonical nucleotides (A, U, G, C) is used with the 2′-OH and 3′-OH groups constrained to prevent the formation of hydrogen bonding between these two groups. Two conformers corresponding to two different orientations of the 2AI bridging group are used for the linker (Me-p-2AI-p-Me). The intermolecular constraint is imposed that the sum of the charges of the 5′-OH group of the nucleotide and either of the two methyl groups of the linker is neutral.

Activated Mononucleotide. The optimized structure of the four canonical nucleotides (A, U, G, C) is used with the 2′-OH and 3′-OH groups constrained to prevent the formation of hydrogen bonding between these two groups. Two conformers corresponding to two different orientations of the 2AI bridging group are used for the linker (2AI-p-Me). The intermolecular constraint is imposed that the sum of the charges of the 5′-OH group of the nucleotide and either of the methyl group of the linker is neutral.

Table S1. RESP Charges for 2AI-Bridged Diguanosine (A), Deprotonated Guanosine (B), and Activated Guanosine (C)

(A)

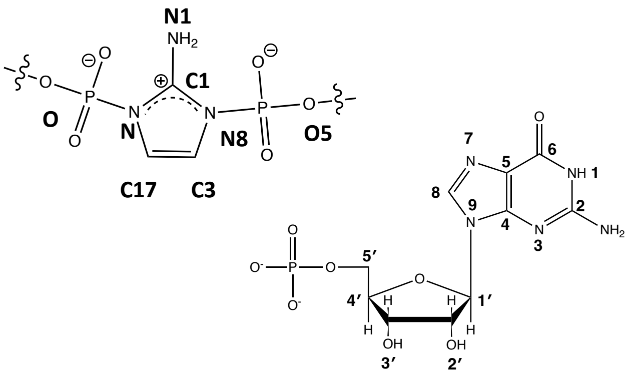

Atom	RESP Charge (e)
**P1**	1.1151
**O1**	-0.7414
**O2**	-0.7414
**P**	1.1182
**O3**	-0.7423
**O4**	-0.7423
**C1**	0.4818
**N11**	-0.9090
**H18**	0.4318
**H2**	0.4318
**N8**	-0.0866
**C17**	-0.2297
**H3**	0.2140
**N**	-0.0872
**C3**	-0.2217
**H4**	0.2114
**O5**	-0.4401
**O**	-0.4404

Atom	RESP Charge (e)
**C1'**	0.0794
**H1'**	0.1731
**C2'**	0.0272
**H2'**	0.1111
**O2'**	-0.6206
**H2T**	0.4329
**C3'**	0.2314
**H3'**	0.0464
**O3'**	-0.6775
**H3T**	0.4411
**C4'**	0.1537
**H4'**	0.1064
**C5'**	0.0547
**H5'1**	0.0650
**H5'2**	0.0650
**O4'**	-0.3710
**C6**	0.5075
**O6**	-0.5439
**C5**	0.1902
**N7**	-0.5733
**C8**	0.1381
**H8**	0.1657
**N9**	0.0154
**C4**	0.1298
**N3**	-0.6085
**C2**	0.7238
**N2**	-0.9080
**H21**	0.3978
**H22**	0.3978
**N1**	-0.5119
**H1**	0.3502

(B)

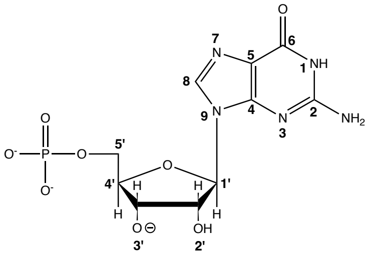

Atom	RESP Charge (e)
**P**	1.1662
**OP1**	–0.7760
**OP2**	–0.7760
**O5′**	–0.4989
**C1′**	0.1071
**H1′**	0.1264
**C2′**	0.0443
**H2′**	0.0825
**O2′**	–0.6582
**HO2′**	0.4268
**C3′**	0.4000
**H3′**	–0.1181
**O3′**	–0.9056
**C4′**	0.1418
**H4′**	0.0606
**C5′**	–0.0326
**H5′**	0.0811
**H5″**	0.0811
**O4′**	–0.4142
**C6**	0.5494
**O6**	–0.5866
**C5**	0.1749
**N7**	–0.6147
**C8**	0.1650
**H8**	0.1618
**N9**	–0.0048
**C4**	0.1558
**N3**	–0.6044
**C2**	0.7481
**N2**	–0.9055
**H21**	0.3779
**H22**	0.3779
**N1**	–0.5843
**H1**	0.3593

Table S1 (continued). RESP Charges for 2AI-Bridged Diguanosine (A), Deprotonated Guanosine (B), and Activated Guanosine (C)

(C)

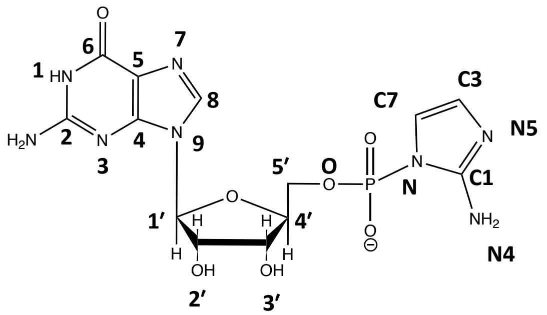

Atom	RESP Charge (e)
P1	1.0251
O1	-0.7415
O2	-0.7415
C1	0.6740
N4	-0.9782
H5	0.3863
H2	0.3863
N5	-0.6817
C7	-0.4176
H3	0.2150
N	-0.0297
C3	0.0472
H4	0.1101
O	-0.4347

Atom	RESP Charge (e)
C1'	0.0768
H1'	0.1736
C2'	0.0278
H2'	0.1115
O2'	-0.6207
H2T	0.4327
C3'	0.2342
H3'	0.0444
O3'	-0.6771
H3T	0.4412
C4'	0.1459
H4'	0.1101
C5'	0.0402
H5'1	0.0691
H5'2	0.0691
O4'	-0.3673
C6	0.5084
O6	-0.5441
C5	0.1886
N7	-0.5736
C8	0.1431
H8	0.1612
N9	0.0150
C4	0.1299
N3	-0.6080
C2	0.7235
N2	-0.9080
H21	0.3977
H22	0.3977
N1	-0.5119
H1	0.3499

Table S2. Summary of Classical Simulations Performed in this Study

Label	Initial Coordinate	Initial Configuration			Time
		3′-OH Protonation State	Coordination to O3′	Coordination to O(pro-S_P)	
**Sim1**	Mittal and Nisler et al.	Protonated	Inner sphere	Inner sphere	100 + 200 ns
**Sim2**	Sim1	Protonated	Outer sphere	Outer sphere	100 + 200 ns
**Sim3**	Sim1	Deprotonated	Outer sphere	Inner sphere	100 + 200 ns
		Coordination to O(pro-S_P)	Coordination to N in 2AI		
**Sim4**	QM/MM product	Inner sphere	Inner sphere		100 ns
**Sim5**	QM/MM product	Inner sphere	Outer sphere		100 + 200 ns
**Sim6**	Sim4	Outer sphere	Inner sphere		100 + 200 ns

Table S3. Energetics (kcal/mol) from QM/MM Adaptive Steered Molecular Dynamics

Step	Barrier Height	Free Energy Change	PMF Source
**React (PO-IS, 3′OH-OS)→ React (PO-IS, 3′OH-IS) [Int (PO-IS, 3′O⁻-IS)]**	18.0	15.7	Figure S5
**React (PO-IS, 3′OH-OS)→ Int (PO-IS, 3′O⁻-OS) [React (PO-IS, 3′OH-OS)′]**	13.7	12.2	Figure S6
**React (PO-IS, 3′OH-OS)→ Int (PO-IS, 3′O⁻-IS)**	21.4	18.5	Figure S7
**React (PO-IS, 3′OH-OS)→ Prod (PO-IS, 3′O-OS)**	16.4	-1.6	Figure S8
**Int (PO-IS, 3′O⁻-IS)→ Prod (PO-IS, 3′O-IS)**	17.1	8.1	Figure S9
**Int (PO-IS, 3′O⁻-IS)→ Prod (PO-IS, 3′O-OS)**	11.6	-28.5	Figure S10

Energetics from QM Cluster Models

Table S4. Energetics from the QM Cluster Models (in Hartree unless otherwise specified)

r^2SCAN-3c (SMD)

React (PO-IS, 3′OH-OS) → Prod (PO-IS, 3′O-OS)	R	TS	P	Barrier (kcal/mol)	Free Energy Change (kcal/mol)
Electronic Energy	-2459.623946	-2459.609214	-2459.638360	9.2	-9.0
DFT-Corrected Free Energy	-2459.269323	-2459.252847	-2459.283858	10.3	-9.1

Int (PO-IS, 3′O⁻-IS) → Prod (PO-IS, 3′O-IS)	R	TS	P	Barrier (kcal/mol)	Free Energy Change (kcal/mol)
Electronic Energy	-2383.184449	-2383.172147	-2383.186198	7.7	-1.1
DFT-Corrected Free Energy	-2382.850996	-2382.836376	-2382.854308	9.2	-2.1

B3LYP-D4/def2-TZVP (SMD)

React (PO-IS, 3′OH-OS) → Prod (PO-IS, 3′O-OS)	R	TS	P	Barrier (kcal/mol)	Free Energy Change (kcal/mol)
Electronic Energy	-2459.832720	-2459.81395	-2459.846565	11.8	-8.7
DFT-Corrected Free Energy*	-2459.478097 (0.354623)	-2459.457583 (0.356367)	-2459.492063 (0.354502)	12.9	-8.8

Int (PO-IS, 3′O⁻-IS) → Prod (PO-IS, 3′O-IS)	R	TS	P	Barrier (kcal/mol)	Free Energy Change (kcal/mol)
Electronic Energy	-2383.383956	-2383.367772	-2383.386333	10.2	-1.5
DFT-Corrected Free Energy*	-2383.050503 (0.333453)	-2383.032001 (0.335771)	-2383.054443 (0.331890)	11.6	-2.5

B3LYP-D4/def2-TZVPD[2] (SMD)

React (PO-IS, 3′OH-OS) → Prod (PO-IS, 3′O-OS)	R	TS	P	Barrier (kcal/mol)	Free Energy Change (kcal/mol)
Electronic Energy	-2459.845799	-2459.826272	-2459.859234	12.3	-8.4
DFT-Corrected Free Energy*	-2459.491176	-2459.469905	-2459.504732	13.4	-8.5

Int (PO-IS, 3′O⁻-IS) → Prod (PO-IS, 3′O-IS)	R	TS	P	Barrier (kcal/mol)	Free Energy Change (kcal/mol)
Electronic Energy	-2383.396126	-2383.379423	-2383.398962	10.5	-1.8
DFT-Corrected Free Energy*	-2383.062673	-2383.043652	-2383.067072	11.9	-2.8

DLPNO-CCSD(T)/CBS (SMD)

React (PO-IS, 3′OH-OS) → Prod (PO-IS, 3′O-OS)	R	TS	P	Barrier (kcal/mol)	Free Energy Change (kcal/mol)
Electronic Energy	-2457.354858	-2457.336952	-2457.374169	11.2	-12.1
DFT-Corrected Free Energy	-2457.000235	-2456.980585	-2457.019668	12.3	-12.2

Int (PO-IS, 3′O⁻-IS) → Prod (PO-IS, 3′O-IS)	R	TS	P	Barrier (kcal/mol)	Free Energy Change (kcal/mol)
Electronic Energy	-2380.962076	-2380.945748	-2380.966900	10.2	-3.0
DFT-Corrected Free Energy	-2380.628623	-2380.609977	-2380.635009	11.7	-4.0

* DFT corrections at the B3LYP-D4/def2-TZVP level of theory are included in parenthesis and applied to all levels of theory.

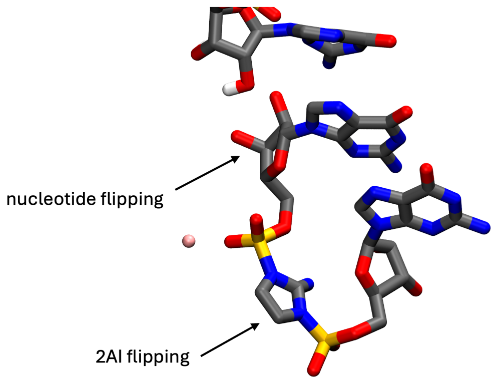

Figure S1. Snapshot of 2AI-bridged dinucleotide and the attacking nucleotide with the 2AI-bridged dinucleotide parameterized using the GAFF2[3] force field and the AM1-BCC charges.[4]

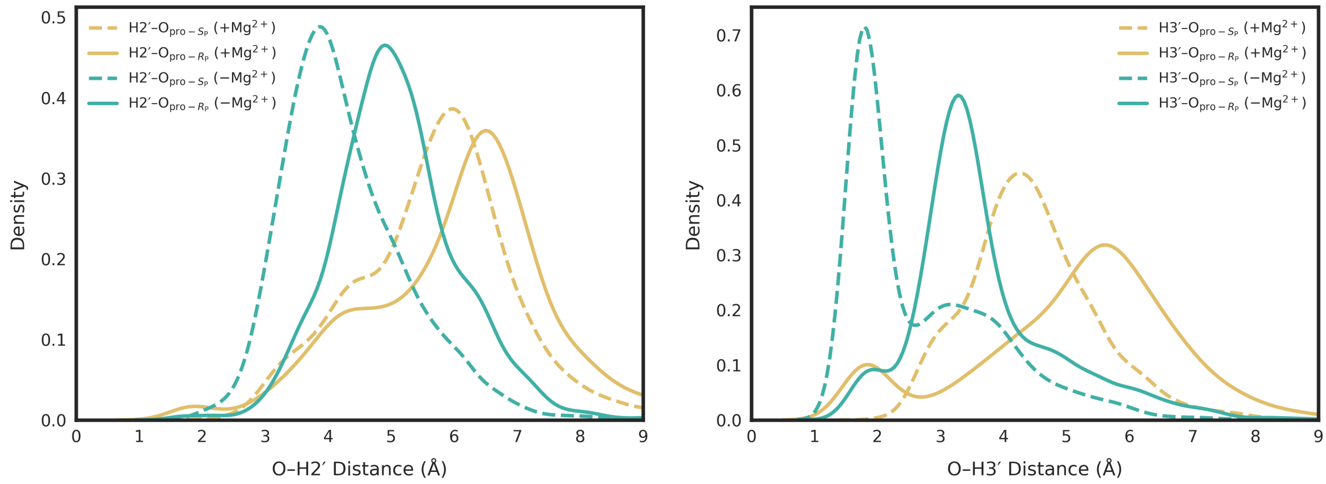

Figure S2. The distance distributions between the proton of the 2′-(left) and 3′-OH (right) group and the pro-S_P (dashed) and pro-R_P (solid) oxygen with (beige; +Mg^{2+}) and without (teal; −Mg^{2+}) Mg^{2+} bound to the pro-S_P oxygen from classical MD simulations.

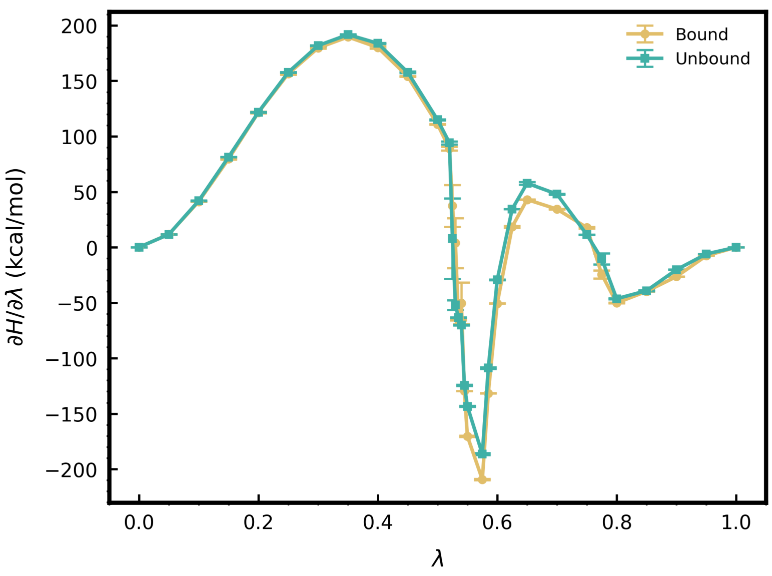

Figure S3. Average of the derivatives of Hamiltonian with respect to alchemical parameter in each window when Mg^{2+} is bound to the pro-S_P oxygen (beige) and in the bulk solution (teal) during thermodynamic integration. The error is calculated with three replicates.

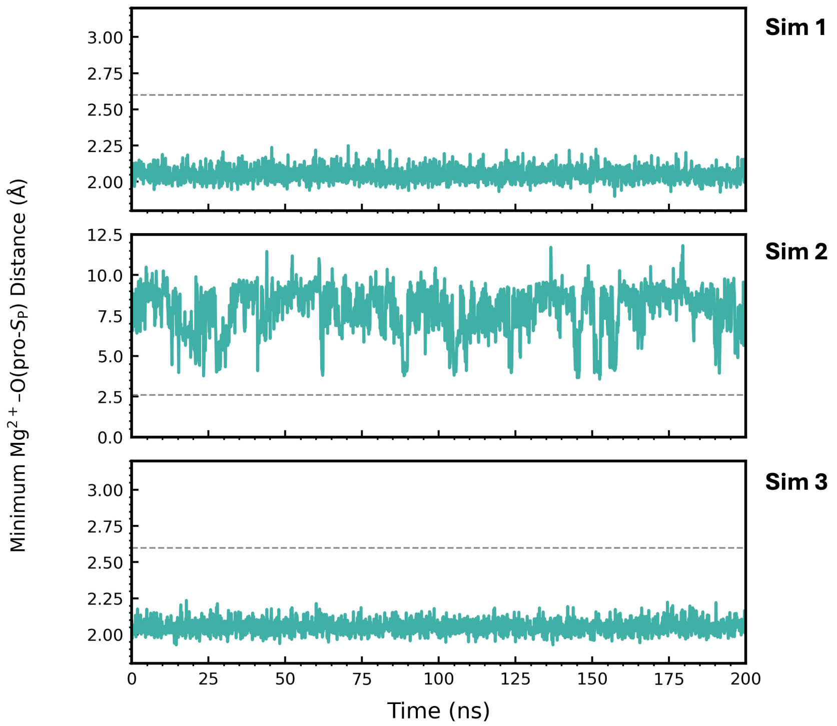

Figure S4. The minimum distance between Mg^{2+} and the pro-S_P oxygen (in Å) in classical equilibrium simulations. The horizontal line represents the distance corresponding to the transition state between the inner- and outer-sphere coordination identified with umbrella sampling at 2.6 Å.

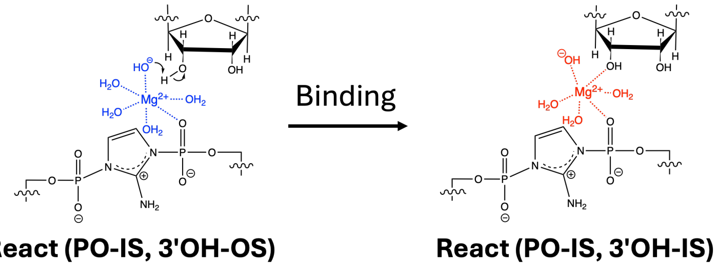

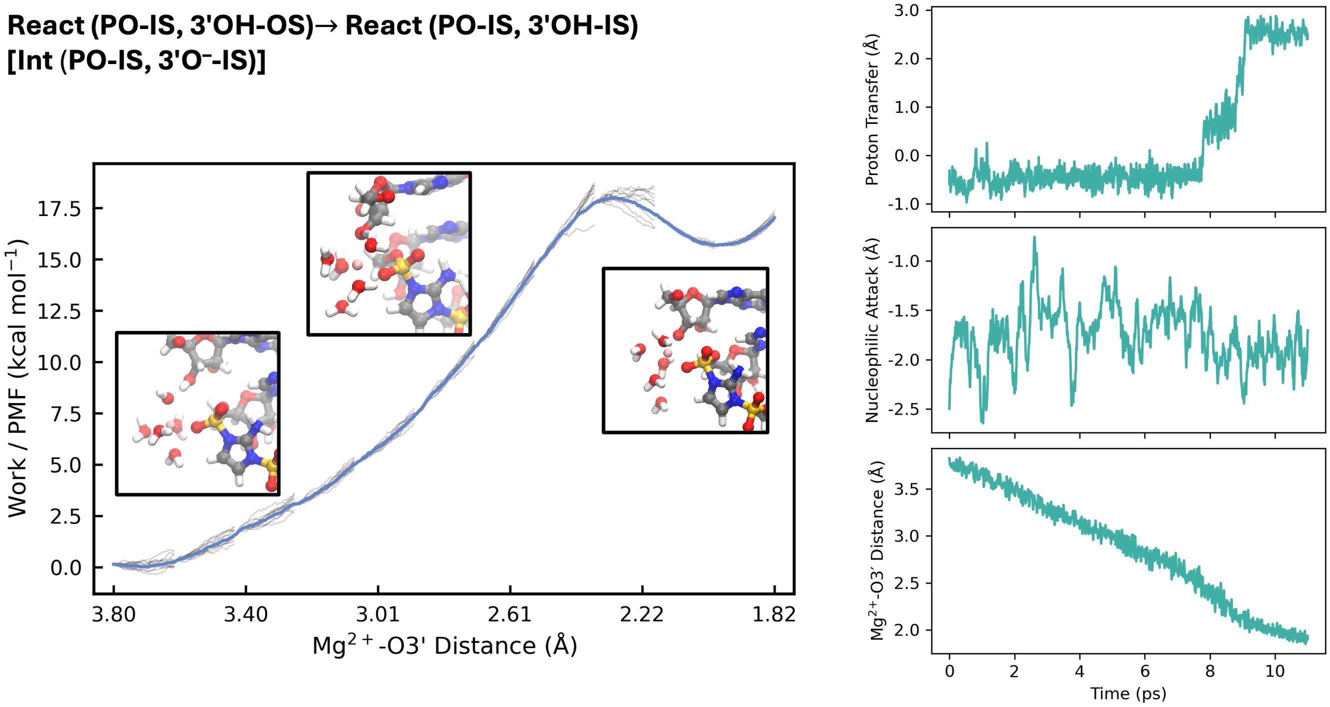

Figure S5. Potential of mean force obtained with adaptive steered molecular dynamics for the pathway **React (PO-IS, 3′OH-OS)** to **React (PO-IS, 3′OH-IS)** according to Scheme 1B. Representative structures of the system are shown along the PMF for the reaction steps. The collective variables of interest (proton transfer, nucleophilic attack, and the Mg^{2+}–O3′ distance) are plotted against time. The corresponding chemical structures are shown at the top of the figure.

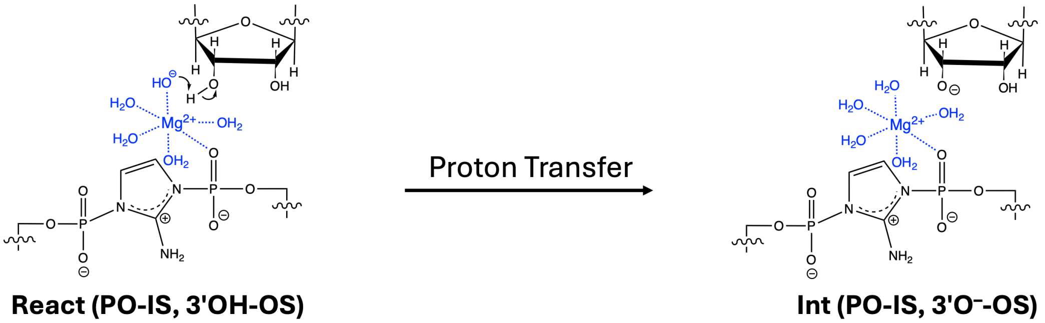

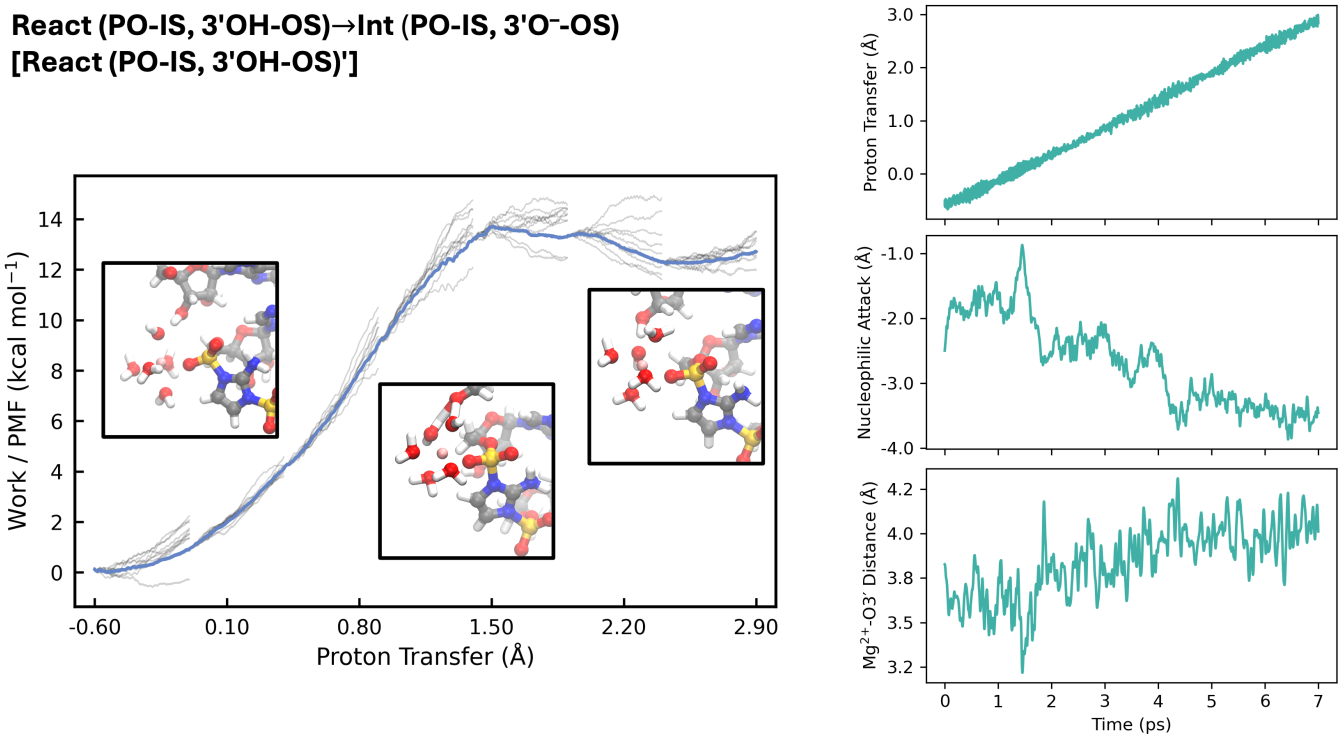

Figure S6. Potential of mean force obtained with adaptive steered molecular dynamics for the pathway **React (PO-IS, 3′OH-OS)** to **Int (PO-IS, 3′O⁻-OS)** according to Scheme 1B. Representative structures of the system are shown along the PMF for the reaction steps. The collective variables of interest (proton transfer, nucleophilic attack, and the Mg^{2+}–O3′ distance) are plotted against time. The corresponding chemical structures are shown at the top of the figure.

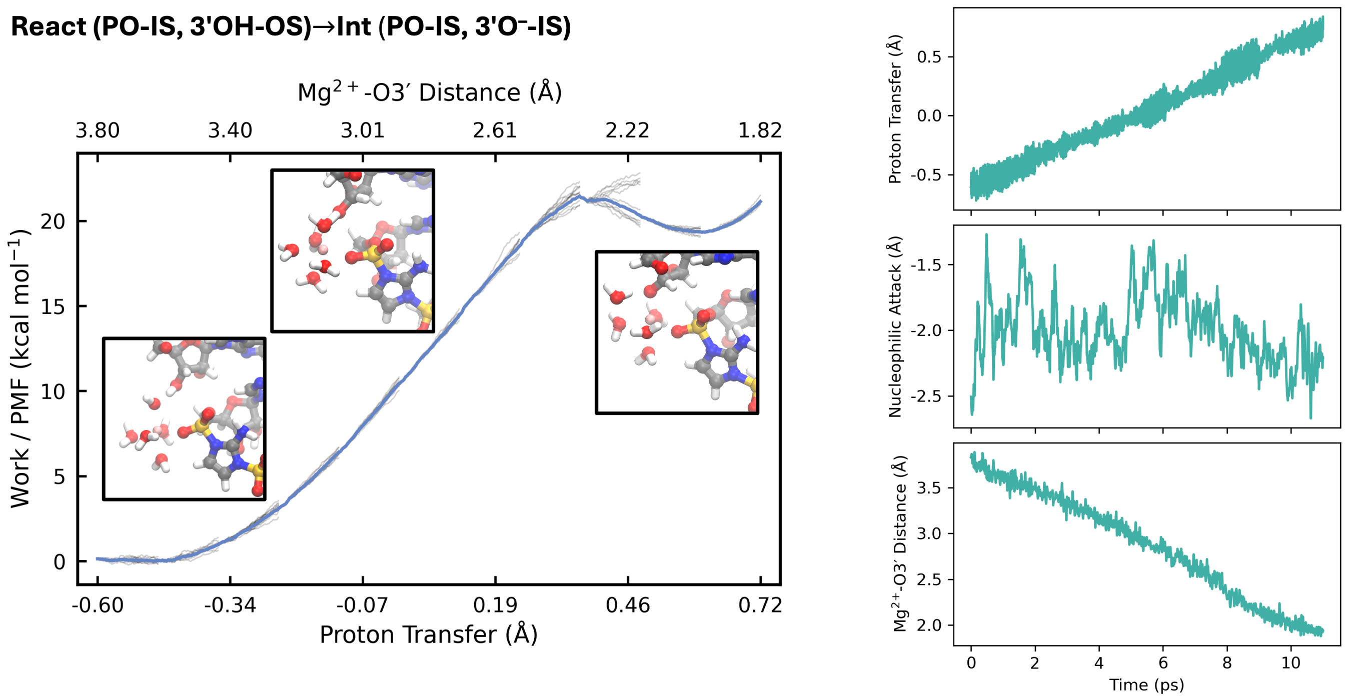

Figure S7. Potential of mean force obtained with adaptive steered molecular dynamics for the pathway **React (PO-IS, 3′OH-OS)** to **Int (PO-IS, 3′O⁻-IS)** according to Scheme 1B. Representative structures of the system are shown along the PMF for the reaction steps. The collective variables of interest (proton transfer, nucleophilic attack, and the Mg^{2+}–O3′ distance) are plotted against time.

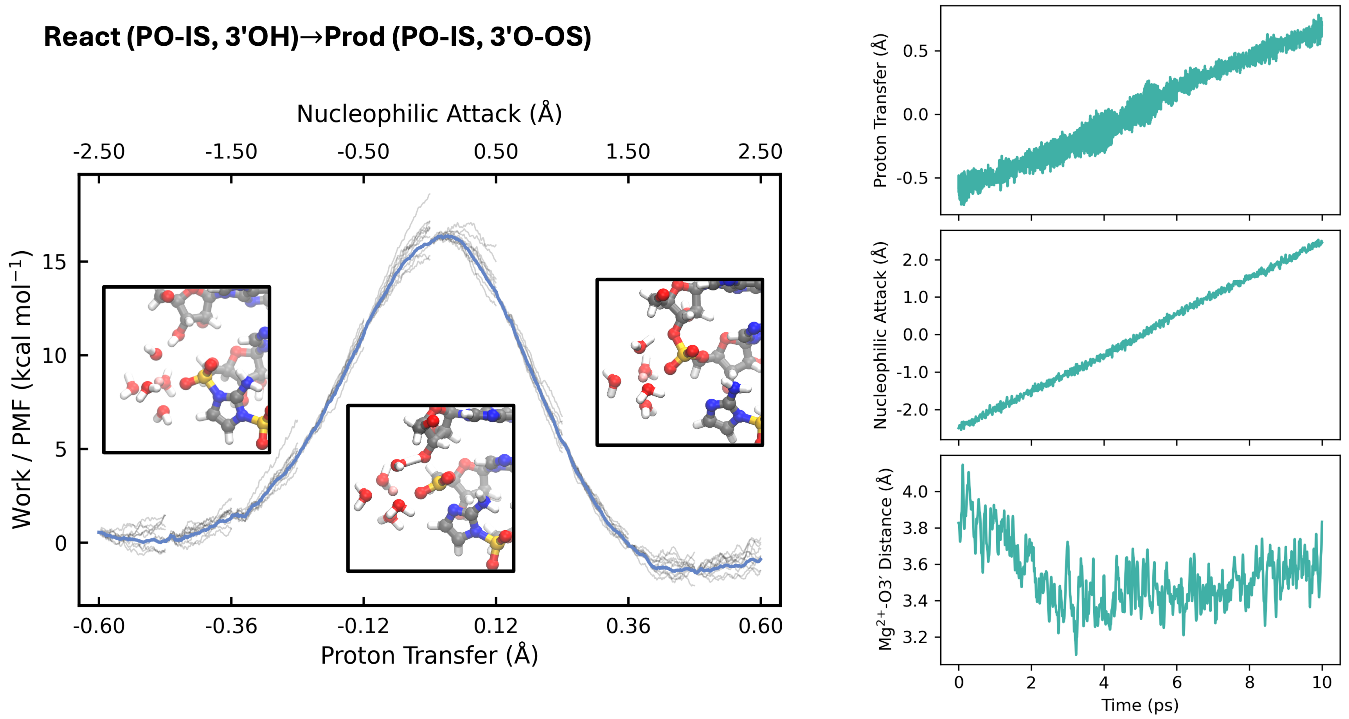

Figure S8. Potential of mean force obtained with adaptive steered molecular dynamics for the pathway **React (PO-IS, 3′OH-OS)** to **Prod (PO-IS, 3′O-OS)** according to Scheme 1B. Representative structures of the system are shown along the PMF for the reaction steps. The collective variables of interest (proton transfer, nucleophilic attack, and the Mg^{2+}–O3′ distance) are plotted against time.

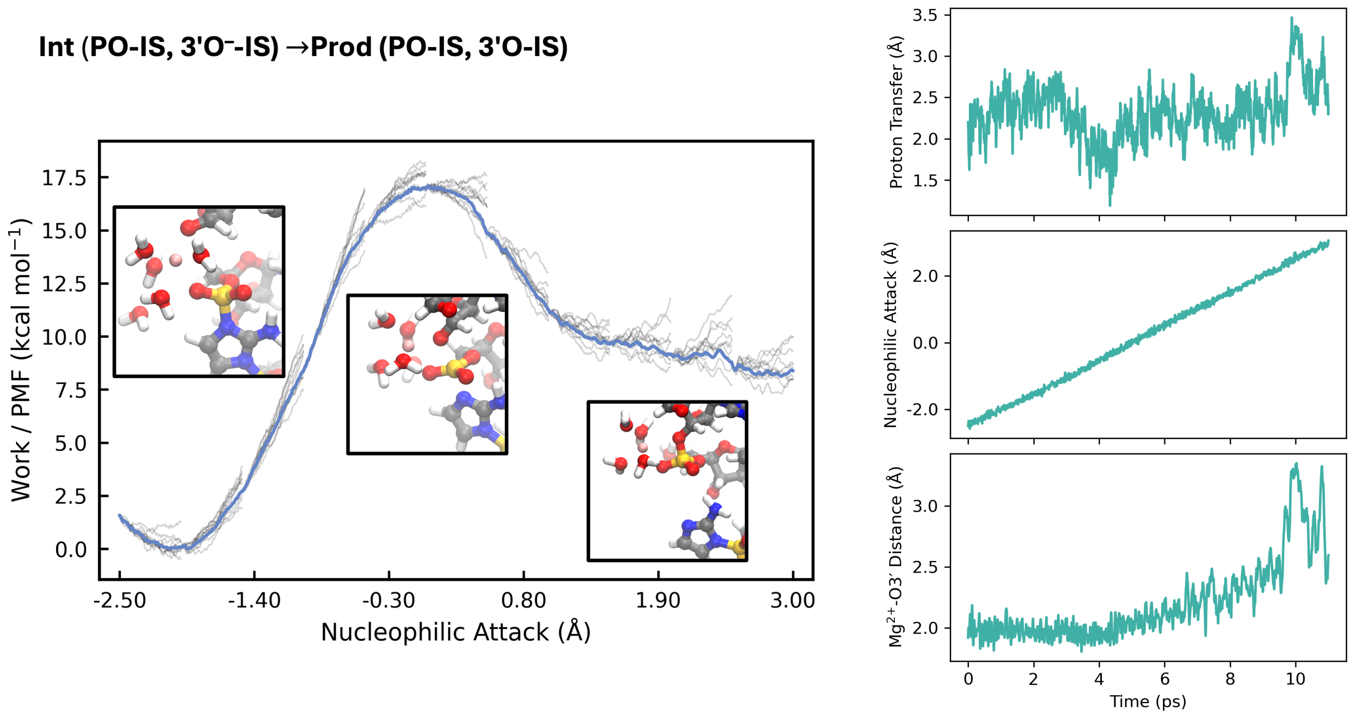

Figure S9. Potential of mean force obtained with adaptive steered molecular dynamics for the pathway **Int (PO-IS, 3′O⁻-IS)** to **Prod (PO-IS, 3′O-IS)** according to Scheme 1B. Representative structures of the system are shown along the PMF for the reaction steps. The collective variables of interest (proton transfer, nucleophilic attack, and the Mg^{2+}–O3′ distance) are plotted against time.

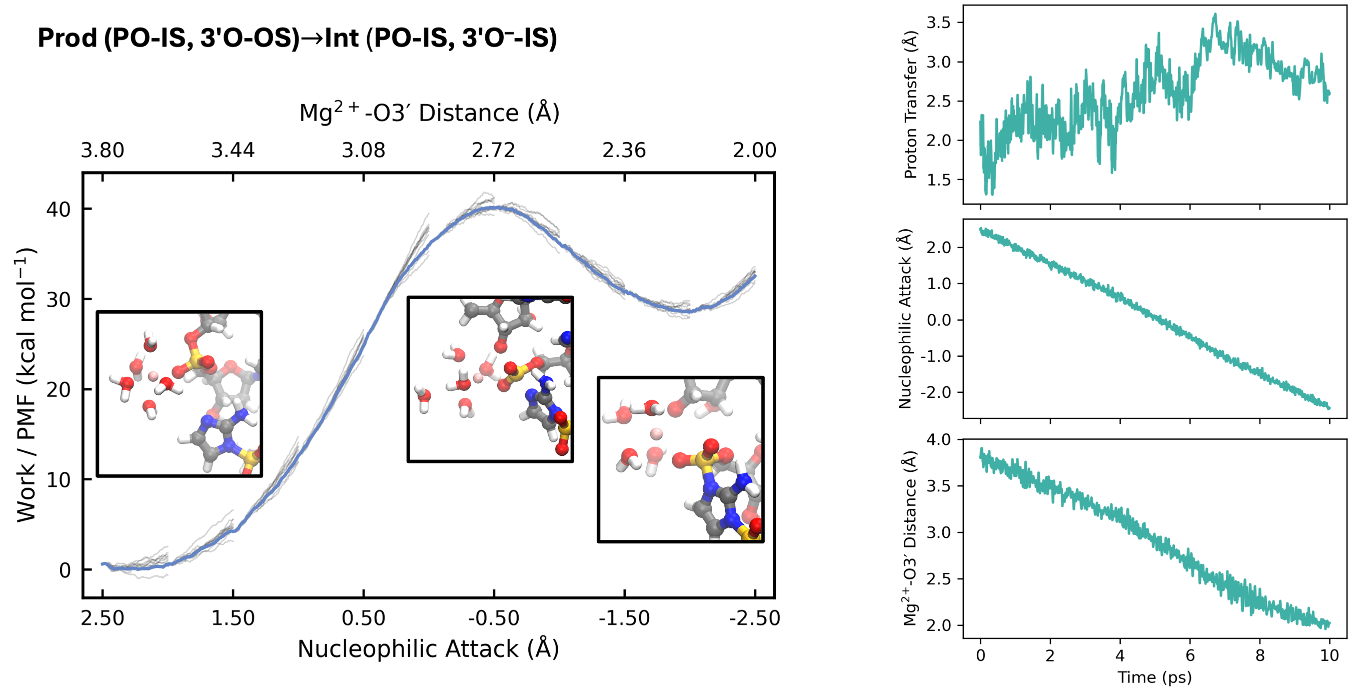

Figure S10. Potential of mean force obtained with adaptive steered molecular dynamics for the pathway **Prod (PO-IS, 3′O-OS)** to **Int (PO-IS, 3′O⁻-IS)** according to Scheme 1B. Representative structures of the system are shown along the PMF for the reaction steps. The collective variables of interest (proton transfer, nucleophilic attack, and the Mg^{2+}–O3′ distance) are plotted against time.

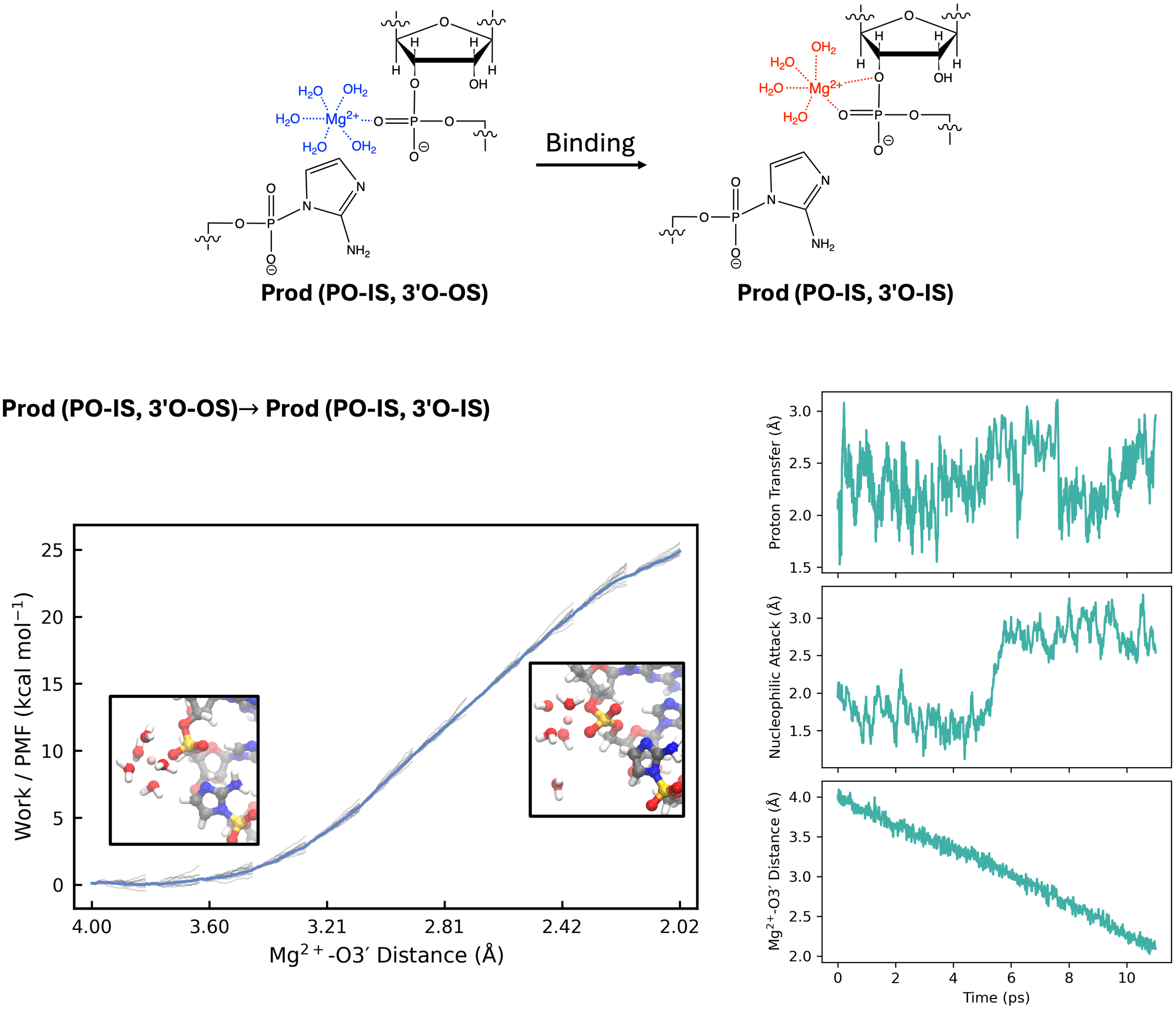

Figure S11. Potential of mean force obtained with adaptive steered molecular dynamics for the pathway **Prod (PO-IS, 3′O-OS)** to **Prod (PO-IS, 3′O-IS)** according to Scheme 1B. Representative structures of the system are shown along the PMF for the reaction steps. The collective variables of interest (proton transfer, nucleophilic attack, and the Mg^{2+}–O3′ distance) are plotted against time. The corresponding chemical structures are shown at the top of the figure.

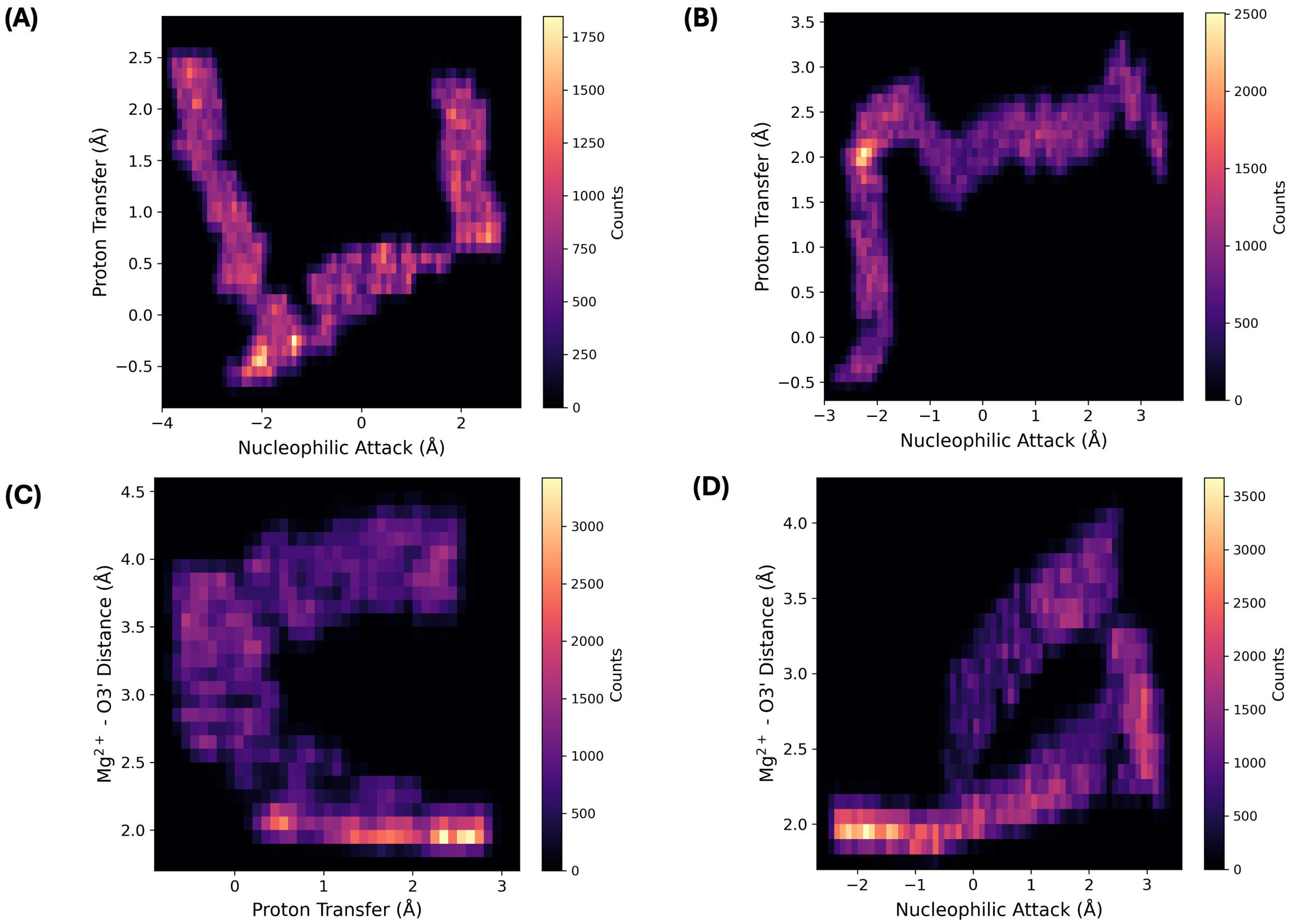

Figure S12. Population histogram of the two-dimensional QM/MM umbrella sampling shown in Figure 4.

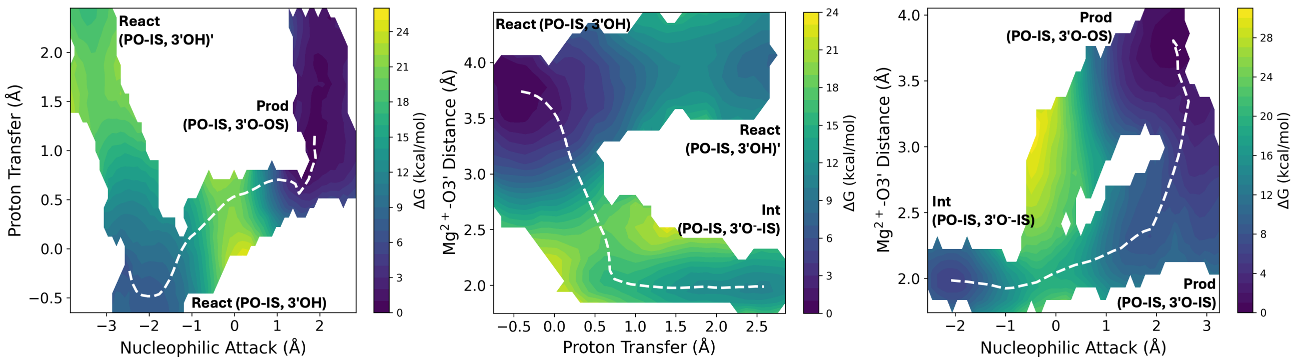

Figure S13. The minimum free energy paths identified with the string method on the two-dimensional free energy landscapes from QM/MM umbrella sampling.

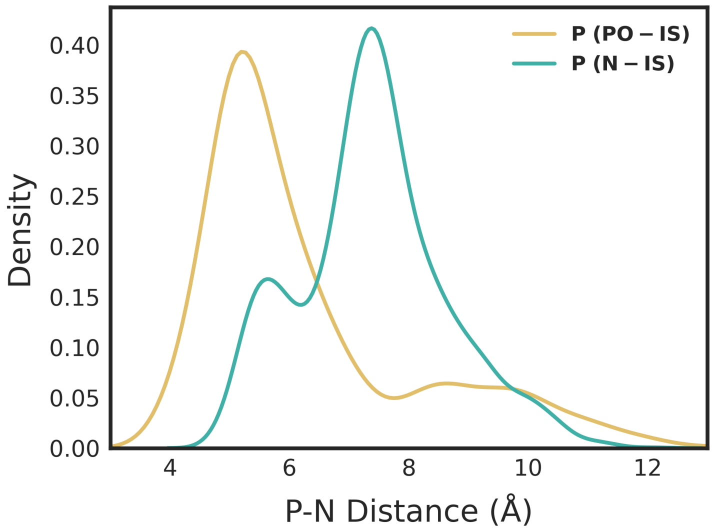

Figure S14. The distance (in Å) between the P atom in the newly formed phosphodiester bond and the unlinked N atom in the 2AI bridging group in the product state **Prod (PO-IS**) and **Prod (N-IS**).

References

(1) Gaussian 09, Revision A.02, M. J. Frisch, G. W. Trucks, H. B. Schlegel, G. E. Scuseria, M. A. Robb, J. R. Cheeseman, G. Scalmani, V. Barone, B. Mennucci, G. A. Petersson, H. Nakatsuji, M. Caricato, X. Li, H. P. Hratchian, A. F. Izmaylov, J. Bloino, G. Zheng, J. L. Sonnenberg, M. Hada, M. Ehara, K. Toyota, R. Fukuda, J. Hasegawa, M. Ishida, T. Nakajima, Y. Honda, O. Kitao, H. Nakai, T. Vreven, J. A. Montgomery, Jr., J. E. Peralta, F. Ogliaro, M. Bearpark, J. J. Heyd, E. Brothers, K. N. Kudin, V. N. Staroverov, R. Kobayashi, J. Normand, K. Raghavachari, A. Rendell, J. C. Burant, S. S. Iyengar, J. Tomasi, M. Cossi, N. Rega, J. M. Millam, M. Klene, J. E. Knox, J. B. Cross, V. Bakken, C. Adamo, J. Jaramillo, R. Gomperts, R. E. Stratmann, O. Yazyev, A. J. Austin, R. Cammi, C. Pomelli, J. W. Ochterski, R. L. Martin, K. Morokuma, V. G. Zakrzewski, G. A. Voth, P. Salvador, J. J. Dannenberg, S. Dapprich, A. D. Daniels, O. Farkas, J. B. Foresman, J. V. Ortiz, J. Cioslowski, and D. J. Fox, Gaussian, Inc., Wallingford CT, 2009.

(2) Rappoport, D.; Furche, F. Property-Optimized Gaussian Basis Sets for Molecular Response Calculations. *J. Chem. Phys.* **2010**, *133* (13), 134105.

(3) Wang, J.; Wolf, R. M.; Caldwell, J. W.; Kollman, P. A.; Case, D. A. Development and Testing of a General Amber Force Field. *Journal of Comput. Chem.* **2004**, *25* (9), 1157–1174.

(4) Jakalian, A.; Jack, D. B.; Bayly, C. I. Fast, efficient generation of high-quality atomic charges. AM1-BCC model: II. Parameterization and validation. *J. Comput. Chem.* **2002**, *23* (16), 1623–1641.
